# A surface-exposed cardiolipin synthase provides an unexpected paradigm for maintaining the Gram-negative outer membrane

**DOI:** 10.1073/pnas.2524588123

**Published:** 2026-01-22

**Authors:** Carmen M. Herrera, Lucas M. Demey, Courtney K. Ellison, M. Stephen Trent

**Affiliations:** ^a^Department of Infectious Diseases, College of Veterinary Medicine, University of Georgia, Athens, GA 30602; ^b^Department of Microbiology, College of Art and Sciences, University of Georgia, Athens, GA 30602

**Keywords:** cardiolipin, outer membrane, *Acinetobacter*, lipoprotein, phospholipids

## Abstract

Gram-negative bacteria have long been thought to confine primary glycerophospholipid synthesis to the inner membrane (IM). Here, we challenge this paradigm by identifying ClsO, an outer membrane (OM) lipoprotein from *Acinetobacter baumannii* that functions as a cardiolipin synthase. In contrast to canonical cardiolipin synthases, which are restricted to the IM, ClsO is surface-exposed and deposits cardiolipin into the outer leaflet of the OM, colocalizing with *Acinetobacter* lipooligosaccharide. Loss of ClsO sensitizes cells to membrane disrupting agents, such as bile salts, and key antibiotics, implicating ClsO in OM barrier integrity. ClsO activity increases when mislocalized glycerophospholipids accumulate at the cell surface, such as in cells lacking Mla (maintenance of lipid asymmetry) system, consistent with a surface-active site mechanism. Engineering mutations in ClsO that prevent its trafficking to the OM was found to abolish nearly all enzymatic activity. Remarkably, we found that ClsO homologs are widespread across Proteobacteria and confirmed several to be functional cardiolipin synthases, indicating a conserved strategy of surface lipid remodeling. These findings redefine the spatial organization of lipid metabolism in Gram-negative bacteria and reveal an adaptive mechanism to maintain OM integrity when membrane asymmetry is compromised.

## Significance

The outer membrane of Gram-negative bacteria is a unique structure that protects cells from environmental stress and blocks the entry of many antibiotics. Understanding how bacteria build and maintain this membrane is essential for combating multidrug-resistant infections. We identify a conserved enzyme that synthesizes cardiolipin, a specialized lipid, directly at the bacterial surface. This finding challenges the long-held view that glycerophospholipid synthesis is restricted to internal membranes and reveals how bacteria remodel their surface to preserve its barrier function.

Gram-negative bacteria possess a distinctive diderm cell envelope architecture, composed of an inner membrane (IM) and an outer membrane (OM), separated by the periplasmic space containing a thin layer of peptidoglycan (1, 2). This complex structure provides a selective barrier that protects the cell from environmental threats while allowing nutrient exchange, environmental sensing, and signal transduction (3). The IM of Gram negatives is a typical bilayer composed of glycerophospholipids (GPLs). In contrast, the archetypal OM is an asymmetric bilayer: its inner leaflet resembles the IM in lipid composition, while its outer leaflet is composed primarily of lipopolysaccharide (LPS), a glycolipid that plays a critical role in OM integrity (1, 2). In many organisms, LPS is replaced with LOS or lipooligosaccharide, a truncated form of LPS that has fewer sugars as it lacks the lengthy O-antigen domain found in LPS.

Our current understanding holds that both LPS/LOS and GPLs are synthesized on the cytoplasmic side of the IM (2, 4). Following synthesis, these lipids are transported across the periplasm to the OM via specialized transport systems. LPS/LOS is moved by the conserved Lpt (LPS/LOS transport) pathway, which shuttles the molecule in an energy-dependent manner from the IM to the outer leaflet of the OM (5). In this process, the Lpt transporter deposits LPS/LOS directly into the outer leaflet of the membrane helping to establish OM asymmetry. More recently, it was determined that members of the AsmA-like protein family facilitate the transfer of GPLs from the IM to the OM across the aqueous periplasm (6–8).

**Fig. 1.**
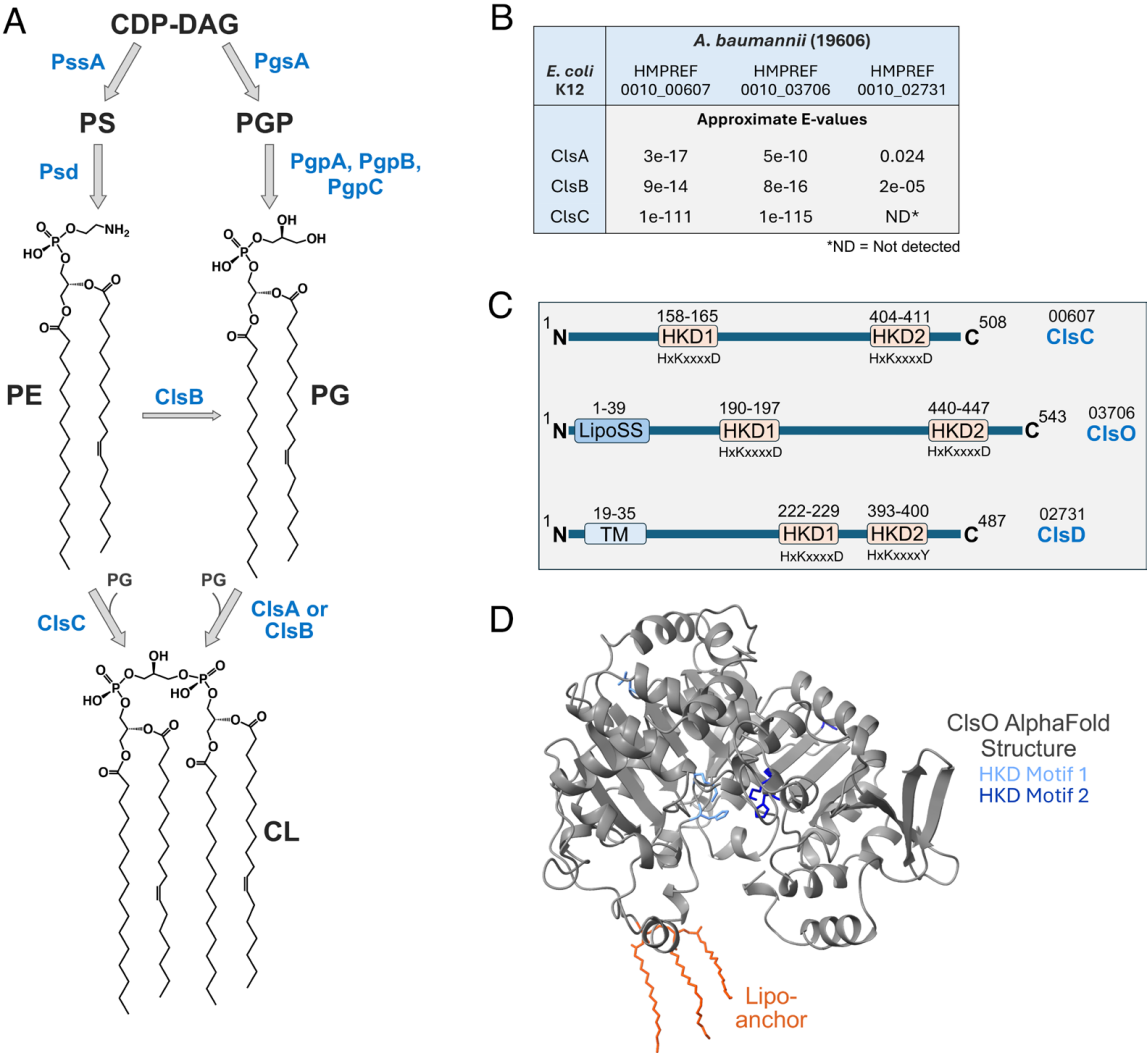
Biosynthetic pathway leading to production of CL and examination of *Acinetobacter baumannii* CL synthases. (*A*) Panel *A* shows the overall pathway for GPL synthesis in *E. coli.* During GPL synthesis, CDP-DAG serves as a donor of phosphatidyl moieties for the synthesis of key GPL precursors. PS is generated by the phosphatidylserine synthase, PssA, and PGP is formed by the phosphatidylglycerol phosphate synthase, PgsA. PS and PGP are then rapidly processed by additional enzymes. PS is decarboxylated by phosphatidylserine decarboxylase (Psd) generating PE, the most predominant GPL in most Gram-negative bacteria. For synthesis of PG, PGP is quickly dephosphorylated by one of several phosphatases (PgpA, B, or C). Finally, CL is synthesized by one of three CL synthases (Cls). In *E. coli*, ClsA and ClsB catalyze the condensation of two PG molecules to form CL, whereas ClsC uses one PG and PE molecule. In *E. coli*, ClsB can also catalyze the conversion of PE to PG at low levels. (*B*) BLASTp scores comparing *E. coli* CL synthases to putative CL synthases of *A. baumannii*. Approximate E-values are shown. *Acinetobacter* ClsO is most closely related to *E. coli* ClsC. See *SI Appendix*, Table S1 for more details. (*C*) Schematic highlighting structural features of the putative CL synthases of *A. baumannii*, including transmembrane (TM) domains, lipoprotein signal peptides (LipoSS), and the two HKD motifs commonly found in CL synthases. The presence or absence of TM domains, signal peptides, and protein motifs were evaluated using DeepTMHMM, Signal P 6.0, and ProSite, respectively. (*D*) Alphafold 3.0 ribbon structure of *A. baumannii* ClsO. Structure indicates the triacylated lipo-anchor (orange) as well as the two HKD motifs in light (HKD1) and dark (HKD2) blue.

In most bacteria, GPL synthesis begins with the precursor cytidine diphosphate-diacylglycerol (CDP-DAG), which serves as a central donor of phosphatidyl groups for the generation of key membrane phospholipids (1, 9) (Fig. 1*A*). From CDP-DAG, two major biosynthetic branches emerge: one leading to phosphatidylserine (PS) via PssA and the other to phosphatidylglycerol-phosphate (PGP) via PgsA. PS is rapidly decarboxylated by Psd to form phosphatidylethanolamine (PE), the most abundant GPL in wild-type *Escherichia coli* (~70%). Much like PS, PGP is a short-lived intermediate and is quickly dephosphorylated by PgpA/B/C to yield phosphatidylglycerol (PG), which accounts for ~20% of total membrane GPLs (1, 9) (Fig. 1*A*).

The last of the “major” GPL species is cardiolipin (CL). CL is synthesized through the condensation of two PG molecules or, in some cases, one PG and one PE molecule, depending on the specific CL synthase (Fig. 1*A*). In wild-type *E. coli*, three known CL synthases (ClsA, ClsB, and ClsC) localize to the IM and perform CL biosynthesis on the cytoplasmic face in wild-type cells. There has been some controversy regarding the membrane topology of ClsA (10, 11); however, a more recent report demonstrated ClsA-dependent CL synthesis occurs in the cytoplasmic compartment of wild-type *E. coli* under normal growth conditions (12). Notably, under certain physiological states, the ClsA active site can reorient toward the periplasm, raising the possibility of limited CL synthesis on the periplasmic side of the IM (12). Although CL is the least abundant (~5%) of the three major GPLs during logarithmic growth, it has been shown to support multiple aspects of cellular membrane dynamics including the subcellular distribution of proteins, the stability of protein complexes, and the regulation of enzymatic activity (11, 13–19).

Much like *E. coli*, the multidrug-resistant pathogen *A. baumannii* encodes three putative CL synthases. Two of these enzymes are predicted to localize to the IM and contain no signal peptides, consistent with existing models of membrane lipid biosynthesis. Unexpectedly, we identified the third enzyme as a lipoprotein localized to the OM. For this reason, we now refer to the enzyme as ClsO where “O” indicates its OM topology. ClsO is most like ClsC of *E. coli* and is predicted to utilize one molecule each of PG and PE to generate CL. More remarkably, we found that ClsO is a surface-exposed lipoprotein, suggesting that CL synthesis can occur in the outer leaflet of the OM—where it would colocalize with LOS as *A. baumannii* is an LOS-producing organism. ClsO was responsible for the bulk of CL production at both logarithmic and stationary phases of growth. Surprisingly, ClsO activity was found to be dependent on the site of membrane localization. Engineered ClsO variants that were localized to the periplasmic face of the IM showed a major reduction in enzymatic activity. Finally, *clsO* mutants were sensitive to detergents, bile salts, and certain antibiotics suggesting that production of CL at the bacterial surface is important for OM integrity and bacterial fitness.

Comparative genomic analyses across a broad range of Gram-negative organisms found that homologs of ClsO are widespread among Proteobacteria, indicating that this noncanonical lipid biosynthetic strategy may be conserved across diverse species. Expression of ClsO homologs from multiple organisms in a CL-deficient *E. coli* strain resulted in robust CL production supporting bioinformatic analyses. These findings expand our understanding of membrane lipid biosynthesis, challenge the traditional compartmentalization of lipid metabolic pathways, and suggest alternative mechanisms for envelope lipid diversification and function in Gram-negative organisms.

## Results

### *A. baumannii* Encodes Three Putative CL Synthases, Including a Lipoprotein Homolog.

*A. baumannii* is a highly drug-resistant organism that is considered a top priority pathogen of concern (20). Compared to the model organism *E. coli*, work from our group and from our colleagues has demonstrated that the bacterium possesses unique characteristics regarding the assembly and regulation of its cell envelope structure (21–26). Building on this foundation, we sought to investigate GPL biogenesis in this organism focusing on the synthesis of CL. Despite its lower abundance, we and others have demonstrated that CL plays critical roles in membrane organization, stress responses, and protein complex stabilization (13, 17, 18, 27).

Three putative CL synthases were detected in the *A. baumannii* 19606 genome and were compared to each of the *E. coli* enzymes using BLASTp (Fig. 1*B* and *SI Appendix*, Table S1). Each protein sequence was analyzed for possible TM domains, signal peptides, and for the HKD (His-Lys-Asp) motif that is found in these enzymes (Fig. 1*C*). All known CL synthases belong to the phospholipase D (PLD) superfamily and are defined by two highly conserved catalytic HKD motifs, characterized by the consensus sequence HxKxxxxD (28). These motifs are positioned on opposite sides of the enzyme and come together to form a catalytic center that mediates transphosphatidylation reactions. In Cls enzymes, the HKD domains catalyze the condensation of phospholipids (typically PG and/or PE) to produce CL and water (28).

Based on these analyses, we chose to refer to HMPREF0010_00607 as ClsC_Ab_ as it has the highest similarity to *E. coli* ClsC (E-value of 1e-111) and, like ClsC_Ec_, has no TM domains (29). HMPREF0010_02731 is most related to *E. coli* ClsB, but we have designated this homolog as ClsD_Ab_ based on two observations. First, 02731 contains a single TM domain that is not found in ClsB_Ec_ nor does it match the two TM domain architecture of ClsA_Ec_ (12, 29). Second, closer inspection of its catalytic domains revealed an atypical substitution in one of the conserved HKD motifs with the second motif harboring a tyrosine in place of the canonical aspartate (Fig. 1*C*). This unusual substitution may suggest a functional divergence and provided further justification for distinguishing this enzyme with a different name. Surprisingly, the final putative CL synthase (HMPREF0010_03706) was found to contain a lipoprotein signal sequence (LipoSS) as predicted by SignalP 6.0 (30) (Fig. 1*C* and *SI Appendix*, Table S2). The predicted AlphaFold structure of the mature protein with its lipid anchor is shown in Fig. 1*D*. This would indicate GPL biogenesis outside of the cytoplasmic compartment. Both BLASTp analysis and comparison of the predicted AlphaFold structures (*SI Appendix*, Fig. S1) of the *E. coli* enzymes suggest 03706 is most similar to *E. coli* ClsC and likely utilizes one molecule of both PG and PE to generate CL. Here, we demonstrate that 03706 is localized to the OM of the cell envelope and, for that reason, we have renamed this protein ClsO where “O” stands for the OM.

### ClsO Is the Dominant CL Synthase in *A. baumannii*.

To determine the individual contributions of ClsC, ClsO, and ClsD to total CL production, we constructed single, double, and triple deletion mutants in *A. baumannii*. Deletion of any single *cls* gene did not impact growth in Luria-Bertani broth (LB) and only a slight growth defect was observed in the triple mutant (*SI Appendix*, Fig. S2). A similar result is seen for *E. coli* lacking all CL synthases when grown in rich media (13). To evaluate GPL synthesis, strains were grown in LB supplemented with ^32^P_i_ to radiolabel newly synthesized GPLs. Following lipid extraction, GPLs were separated by thin-layer chromatography (TLC) on silica gel plates using a solvent system consisting of chloroform, methanol, and acetic acid (65:25:10, v/v/v) and visualized by phosphorimaging.

**Fig. 2.**
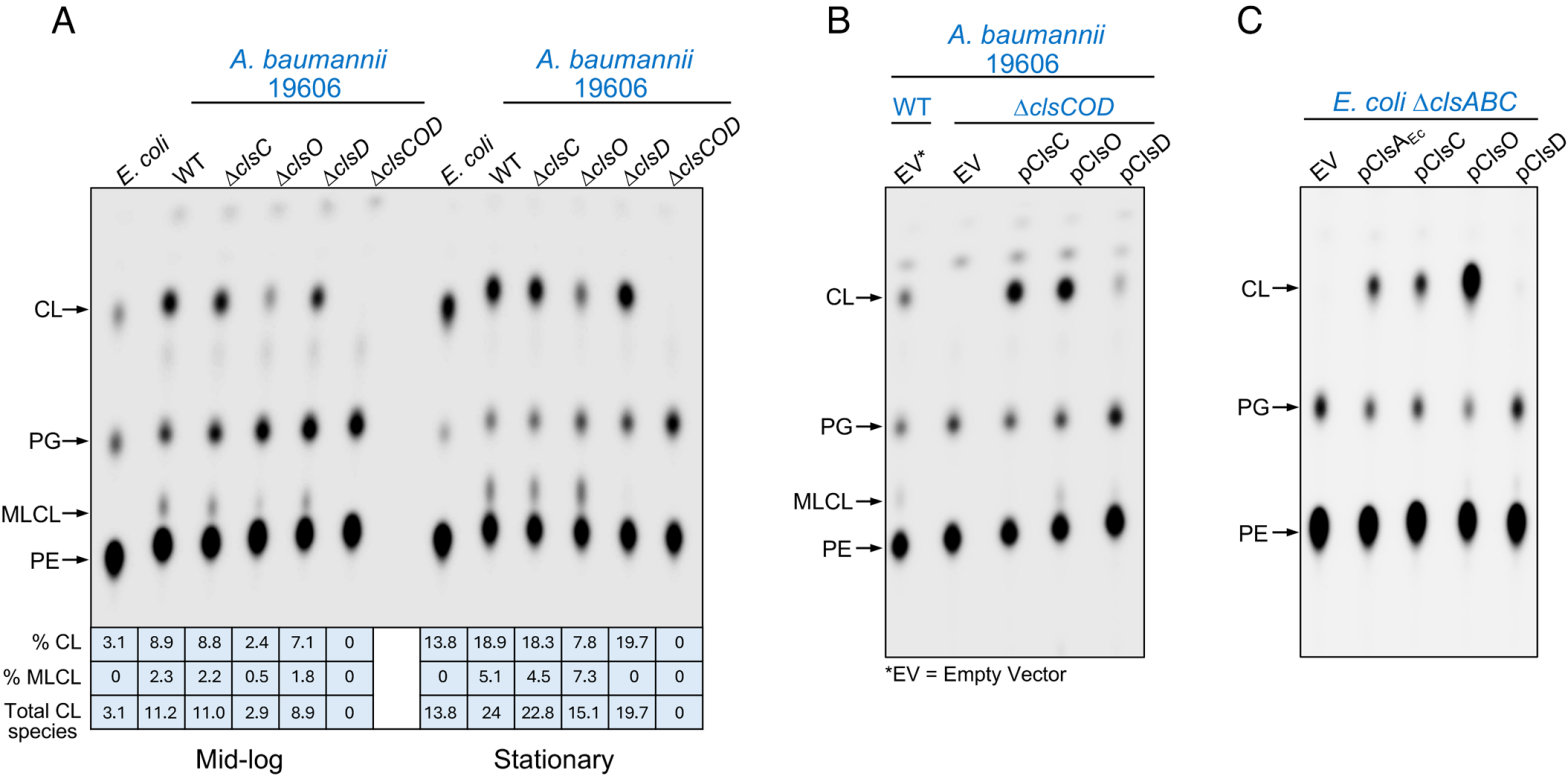
Role of *A. baumannii* CL synthase homologs in GPL synthesis. (*A*) *A. baumannii* 19606 lacking one or more genes encoding putative CL synthases were grown in the presence of ^32^P_i_. Radiolabeled GPLs were isolated, separated by TLC, and visualized by phosphorimaging. The most predominant decrease in CL was observed upon deletion of *clsO*. A total absence of CL was observed when all synthases were deleted (Δ*clsCOD*). The percentage of CL (including mono-lysocardiolipin, MLCL), produced by each strain is indicated. Both wild-type *E. coli* K-12 (strain W3110) and *A. baumannii* were included as controls. Note that wild-type *E. coli* does not produce MLCL. (*B* and *C*) CL production during expression of putative *A. baumannii* synthases in either CL-deficient *A. baumannii* (*B*) or *E. coli* (*C*). In *A. baumannii*, CL synthases were expressed from plasmid pMMB67EH using 50 µM IPTG. For *E. coli*, genes were expressed from plasmid pBAD18 using 0.02% arabinose. Data in all panels are representative of a minimum of three biological replicates.

As reported previously, the major GPLs detected in wild-type were PE, PG, and CL (31–33). Compared to the *E. coli* K-12 control (strain W3110), *A. baumannii* 19606 produced substantially more CL at both mid-log and stationary phase (Fig. 2*A*). As previously reported (34), we also detected monolysocardiolipin (MLCL), a derivate of CL lacking a single acyl chain that is rarely detected in bacterial membranes, including *E. coli*. MLCL represented about 2% of the GPL fraction, further increasing the total amount of CL species in *A. baumannii*. Among single-deletion mutants, loss of *clsO* caused the most pronounced reduction in CL levels in mid-log cultures (>three-fold decrease), whereas deletion of *clsC* or *clsD* showed little to no impact on synthesis. To test for functional redundancy and possible compensatory effects, we analyzed CL levels in double-deletion mutants (*SI Appendix*, Fig. S3). The most severe reductions in CL synthesis were again observed when *clsO* was absent, supporting its role as the dominant CL synthase. Notably, the Δ*clsCOD* triple mutant lacked all detectable CL species, confirming that ClsO, ClsC, and ClsD collectively account for all CL synthase activity in *A. baumannii* (Fig. 2*A* and *SI Appendix*, Fig. S3).

Given the level of strain variation, these results were confirmed in a second strain, *A. baumannii* 5075, that also encodes three CL synthases (*SI Appendix*, Fig. S4*A*). Insertion mutants from the 5075 ordered transposon library (*SI Appendix*, Fig. S4*B*) (35) were verified and changes in GPL production evaluated by TLC (*SI Appendix*, Fig. S4 *C* and *D*). Although strain 5075 makes less CL compared to 19606 during logarithmic growth, the greatest decrease in CL species was still observed upon loss of ClsO (>three-fold decrease).

In *E. coli*, it is well established that CL levels increase during stationary phase coinciding with enhanced expression of ClsB and ClsC (29), suggesting a transcriptionally regulated adaptation to slowed growth or nutrient limitation. How increased CL levels promote cellular fitness in stationary phase, however, is not yet fully understood. To determine if a similar mechanism exists in *A. baumannii*, we performed RNA-seq analysis comparing transcript levels for each CL synthase during log, early stationary, and late stationary phases of growth (*SI Appendix*, Table S3). For the *E. coli* K-12 control, we observed the expected increase in transcripts for both *clsB* and *clsC*. For example, there was a ~9-fold increase in *E. coli clsB* expression in late stationary phase compared to logarithmic growth. However, while the synthesis of CL and MLCL more than doubles in *Acinetobacter* in stationary phase (Fig. 2*A* and *SI Appendix*, Figs. S3 and S4*D*), transcript levels of *clsC*, *clsD*, and *clsO* did not change appreciably. This implies that CL accumulation in *A. baumannii* is regulated posttranscriptionally, perhaps through altered enzyme localization or stability rather than via transcriptional control.

Complementation studies reinforced the role of each CL synthase (Fig. 2*B*). Expression of either ClsO_Ab_ or ClsC_Ab_ in Δ*clsCOD* using the IPTG-inducible plasmid pMMB67EH restored CL synthesis to more than wild-type levels. However, expression of ClsD_Ab_ could only partially restore synthesis. Next, we determined if the *Acinetobacter* enzymes could complement an *E. coli* CL-deficient strain, Δ*clsABC,* using the arabinose-inducible plasmid pBAD18 (Fig. 2*C*). ClsO_Ab_ showed robust activity even surpassing the activity of *E. coli* ClsA during high levels of expression. The activity of ClsC_Ab_ was like that of ClsA_Ec_, but heterologous expression of ClsD_Ab_ showed little activity highlighting its functional conservation and efficiency across species.

### ClsO Is an OM-Localized CL Synthase.

**Fig. 3.**
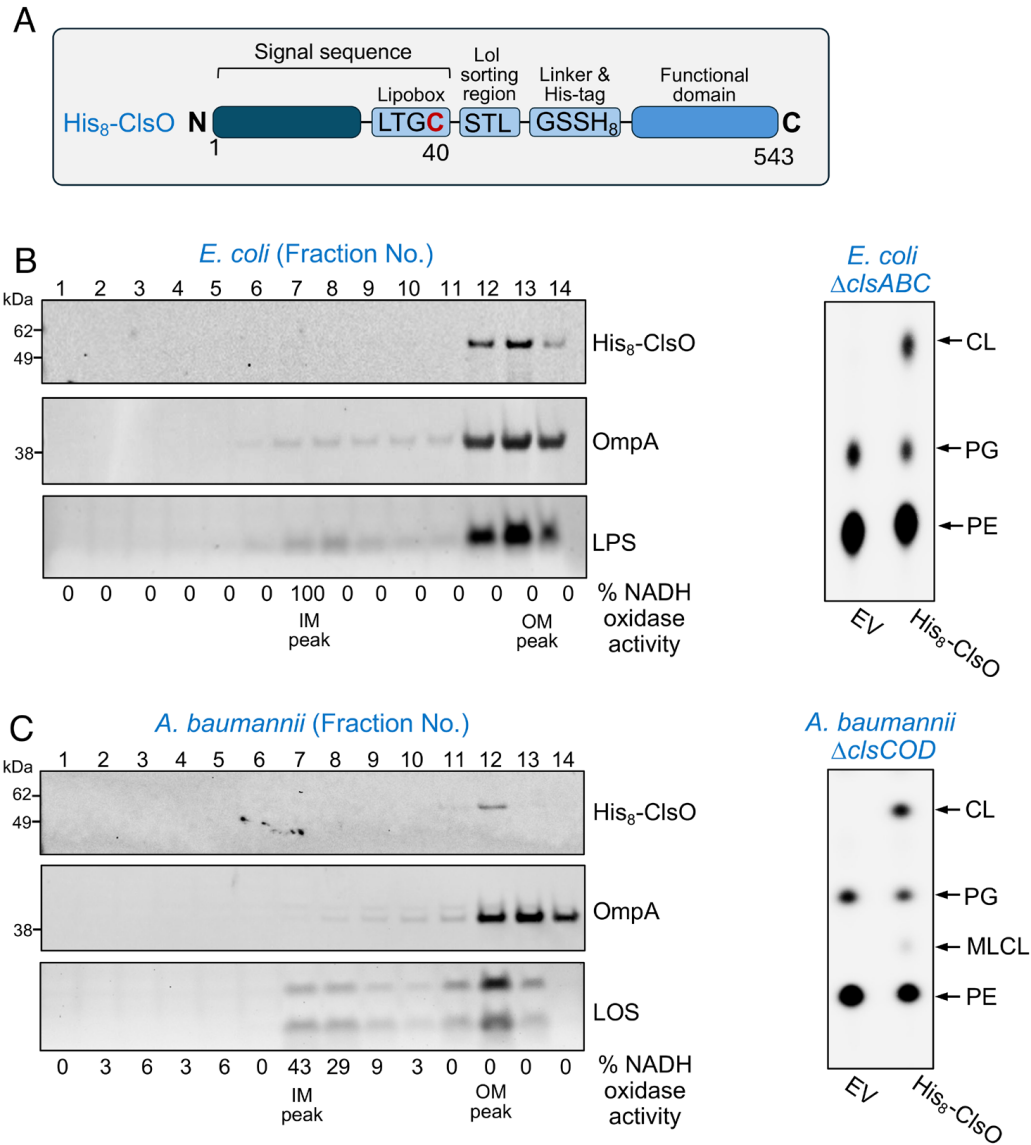
The lipoprotein ClsO is an OM CL synthase. (*A*) Schematic highlighting the lipoprotein signal peptide of ClsO containing the lipobox motif and the putative Lol sorting region. The N-terminal, lipidated cysteine residue of the mature protein is shown in red. To visualize the protein for membrane separation studies, a short linker (GSS) and His_8_-tag was inserted just after the Lol sorting region (STL). (*B* and *C*) OM and IM fractions from either CL-deficient *E. coli* (*B*) or CL-deficient *A. baumannii* (*C*) expressing His_8_-tagged ClsO from plasmid pMMB67EH were separated by isopycnic sucrose density gradient centrifugation. For each fraction, the presence of His_8_-ClsO was evaluated by western blot and for IM and OM markers. OM fractions were determined by detection of either LPS (*E. coli*) or LOS (*A. baumannii*) by SDS-PAGE and for the presence of the OM protein OmpA by western blot. IM peak fractions were determined by NADH oxidase activity (% of total activity across gradient). Enzymatic activity of His_8_-ClsO was verified by evaluating production of CL in both genetic backgrounds following ^32^P_i_ labeling and TLC analysis of radiolabeled GPLs. Data in all panels are representative of a minimum of three biological replicates.

Given ClsO has a lipoprotein signal peptide (residues 1 to 39 of the preprotein) (Fig. 1*C*), we wanted to determine if ClsO localizes to the IM or the OM. We began in *E. coli*, as separation of IM and OM fractions using isopycnic sucrose density gradients is well established for this organism (7, 13, 36, 37). Initially, ClsO was expressed with a C-terminal His_8_-tag (*SI Appendix*, Fig. S5*A*) to avoid complications with the signal peptide. However, this resulted in almost complete loss of activity (*SI Appendix*, Fig. S5*B*). AlphaFold predictions indicated the His_8_-tag likely occluded the enzymatic active site. Next, a N-terminally tagged construct was generated with a short linker and His-tag (GSSH_8_) placed after the cleavable N-terminal signal peptide following the predicted Lol (localization of lipoproteins) sorting region (Fig. 3*A*). This region determines whether the Lol system, an essential protein trafficking pathway, engages with the lipoprotein for transport and insertion into the OM. Expression of N-terminally tagged ClsO from plasmid pMMB67EH in CL-deficient *E. coli* resulted in substantial CL production (Fig. 3*B*).

Membranes from *E. coli* expressing His_8_-ClsO were separated by isopycnic sucrose density gradients and the identity of OM or IM fractions determined using established membrane markers (Fig. 3*B*) as previously described (7, 13). The presence of ClsO was determined via western blot using anti-His antibodies. OM fractions were defined by the presence of LPS and the β-barrel OM protein, OmpA. IM fractions were identified based on NADH oxidase activity, an enzyme tightly associated with the IM respiratory chain. His-tagged ClsO (MW of ~60 kDa for the mature protein) cofractionated with LPS and OmpA and was clearly separated from NADH oxidase activity, indicating clear OM localization. Although C-terminally His-tagged ClsO showed little activity, it was also found in the OM fraction (*SI Appendix*, Fig. S5*B*) ruling out the possibility that our N-terminal tag impacted localization.

ClsO membrane localization was also determined in *A. baumannii* (Fig. 3*C*). Previously, our group and others have shown that standard density gradients used for *E. coli* membrane fractionation are unable to reliably separate *A. baumannii* membranes (33, 38). Cian et al. (38) recently reported that further optimization of the sucrose gradient allows for consistent separation. Using this methodology, along with some additional modifications (*Materials and Methods*), we separated the IM and OM fractions of *A. baumannii* expressing His_8_-ClsO. Membrane markers showed that adequate separation was achieved and that ClsO again cofractionated with the OM markers, OmpA and LOS (Fig. 3*C*). The two LOS bands are expected as *A. baumannii* makes two major LOS species, one that is fully extended and one that is not (32, 39). Together, these data support that ClsO is an OM lipoprotein.

### ClsO Is a Surface-Exposed OM Lipoprotein.

**Fig. 4.**
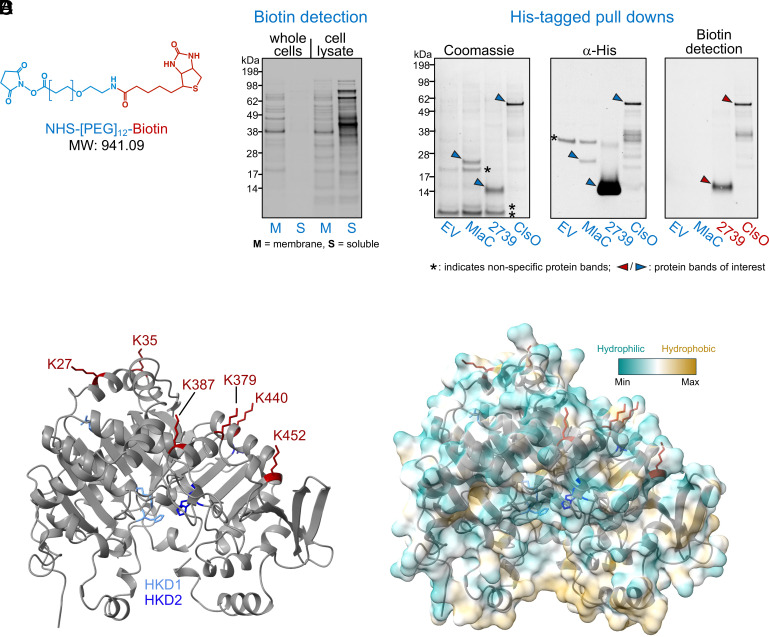
ClsO is an OM surface lipoprotein. (*A*) Structure of NHS-[PEG]_12_-biotin (*N*-hydroxysuccinimide-[polyethylene glycol]_12_ biotin). (*B*) Intact whole cells of wild-type *A. baumannii* or cell lysates were incubated with NHS-[PEG]_12_-biotin to label accessible proteins. The soluble (S) and membrane (M) fractions of each were isolated and the presence of biotinylated proteins detected by western blot. Under the conditions employed, soluble proteins in intact cells were not labeled indicating that NHS-[PEG]_12_-biotin did not cross the OM. Data are representative of three biological replicates. (C) Intact cells individually expressing His-tagged versions of either HMPREF0010_2739 (positive control), MlaC (negative control), or ClsO were exposed to NHS-[PEG]_12_-biotin. Targeted proteins were enriched by anti-His pulldowns and detected by western blot. As expected, the periplasmic protein MlaC (~22 kDa) was not biotinylated. Both ClsO (~60 kDa) and HMPREF0010_2739 (~12 kDa), a previously characterized surface lipoprotein, were both modified indicating surface exposure. Data are representative of three biological replicates. (*D*) The Alphafold ribbon structure and the ribbon structure overlaid with a space-filled hydrophobicity plot of mature ClsO are shown. Lysine residues that were modified with NHS-[PEG]_12_-biotin are displayed in red and the two HKD motifs in light (HKD1) or dark blue (HKD2). Amino acid numbers are those of the mature, untagged, ClsO protein. Hydrophobicity plots were generated using ChimeraX with hydrophilic residues in turquoise and hydrophobic residues in dark gold.

Although once considered to be a rare occurrence, OM lipoproteins from diverse organisms have been shown to be surface-exposed. Furthermore, some surface lipoproteins have enzymatic function, including enzymes utilizing lipid substrates (40, 41). Thus, we wanted to determine if ClsO is surface-exposed using a selective surface biotinylation approach (22, 40, 42) exploiting the reagent NHS-[PEG]_12_-biotin (*N*-hydroxysuccinimide-[polyethylene glycol]_12_ biotin). Under the appropriate conditions, NHS-[PEG]_12_-biotin should specifically label surface-exposed proteins as it is a high-molecular-weight, hydrophilic compound (Fig. 4*A*) that should not easily cross the OM. When whole cells were exposed to NHS-[PEG]_12_-biotin and labeled proteins detected using a streptavidin conjugate, we observed robust labeling of membrane proteins. Soluble proteins, however, were not labeled indicating NHS-[PEG]_12_-biotin fails to efficiently penetrate the OM barrier under the employed conditions (*Materials and Methods*). As expected, if cells were lysed prior to biotinylation both soluble and membrane proteins were labeled (Fig. 4*B*).

To determine if ClsO is surface-exposed, whole cells containing either empty vector or vector expressing His_8_-ClsO were exposed to NHS-[PEG]_12_-biotin. As controls, we monitored labeling of two additional *Acinetobacter* His-tagged proteins, MlaC and HMPREF0010_02739 (2739). 2739 is an OM lipoprotein previously demonstrated to be surface-exposed (22) and served as the positive control for this experiment. MlaC, a soluble periplasmic protein involved in lipid transport (discussed below) (25, 33) operated as the negative control. After biotinylation and quenching of the reaction, cells were lysed and the tagged proteins enriched using HisPur Ni-NTA affinity resin. Targeted proteins were then separated by SDS-PAGE and subjected to Coomassie staining or analyzed by western blot. Compared to the vector control, Coomassie staining showed enrichment of the expected proteins (Fig. 4*C*) as well as nonspecific proteins. A band corresponding to mature, His-tagged ClsO was detected by anti-His antibody with some faster migrating bands that are likely degradation products. The full-length ClsO was also easily detected by streptavidin. As expected, the surface lipoprotein 2739 (MW ~12 kDa) was biotinylated whereas periplasmic MlaC (~22 kDa) was not (Fig. 4*C*).

Next, we assessed the extent of ClsO surface exposure by mapping the sites of biotinylation using proteomics mass spectrometry, which identified six modified, solvent-accessible residues with high confidence. The peptide containing the modified lysine and the position of each residue is shown in *SI Appendix*, Table S4. Mapping these residues onto the predicted AlphaFold structure showed that all modified lysines localized to the hydrophilic face of the protein, on the opposite side of the predicted active site that engages the membrane surface (Fig. 4*D*).

**Fig. 5.**
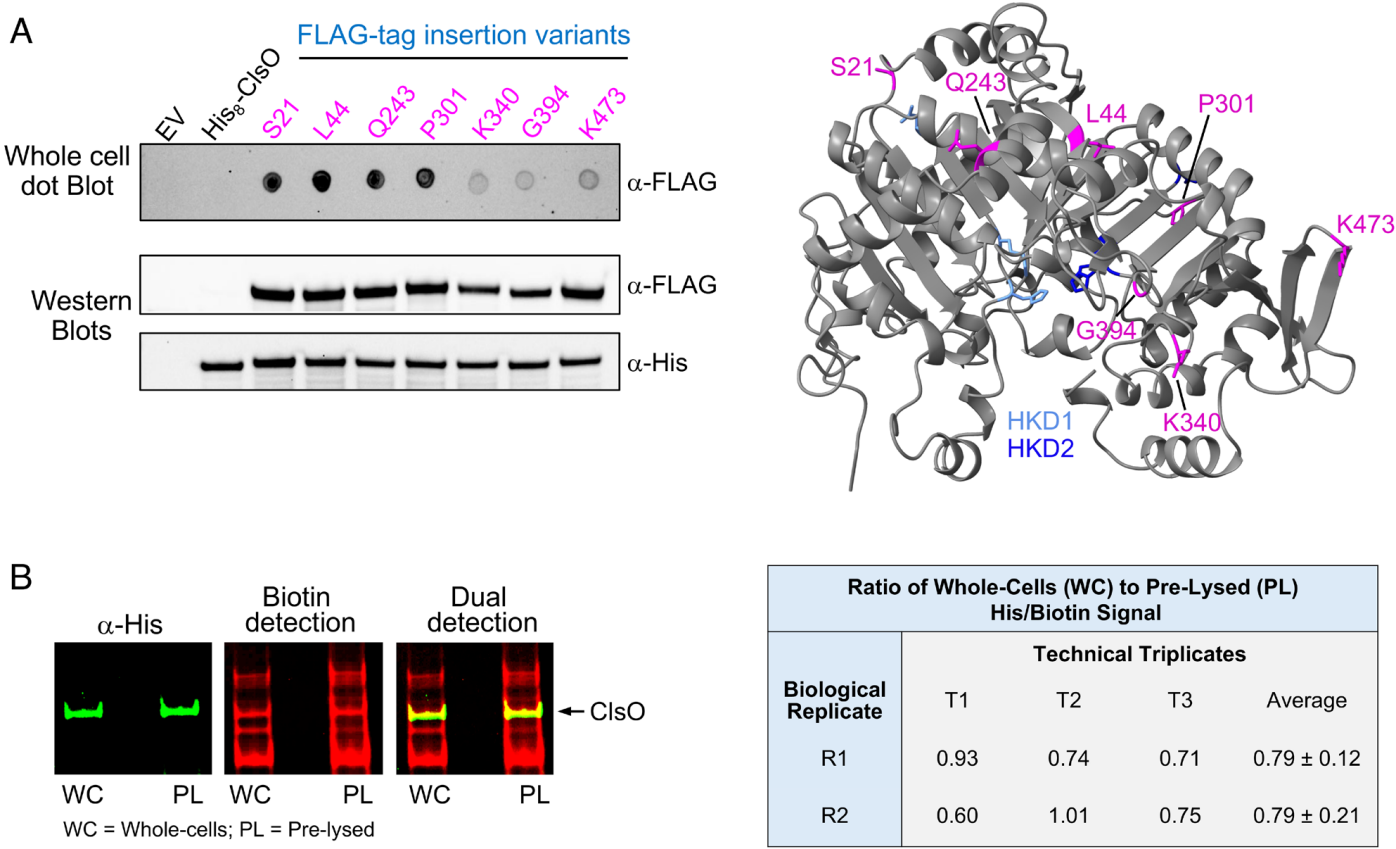
Topological mapping and quantitative assessment of ClsO surface exposure in the OM. (*A*) *Left* panel: Whole cells of wild-type 19606 containing the indicated His_8_-ClsO FLAG-tag (DYKDDDDK) insertion mutants were spotted onto nitrocellulose membrane and probed with an anti-FLAG antibody. Cells containing only the empty vector (EV) or His_8_-ClsO without a FLAG-tag served as controls. To verify ClsO expression, cell lysates were subjected to western blot using both anti-His and anti-FLAG antibodies. Data are representative of three biological replicates. *Right* panel: AlphaFold structure of ClsO indicating the site of FLAG-tag insertions in magenta. The amino acid numbers are those of mature, untagged, ClsO. HKD motifs are indicated. (*B*) *Left* panel: Quantification of ClsO surface exposure by comparing the efficiency of biotin labeling in intact (“whole-cells”) vs. lysed (“pre-lysed”) samples. OM fractions were isolated following NHS-[PEG]_12_-biotin labeling, and ClsO was detected using α-His and streptavidin on dual-channel western blots. Western blot images were acquired and processed under identical conditions for all samples using Bio-Rad Image Lab software. Contrast and brightness were adjusted equally across lanes to facilitate comparison. A representative blot from one technical replicate is shown. *Right* panel: Summary table of His/biotin signal ratios from three technical replicates (T1 to T3) across two biological replicates (R1 and R2). Each value reflects the ratio of His/biotin signal in whole cells vs. the pre-lysed control for the same technical replicate. Ratios near 1.0 indicate similar labeling efficiency between conditions, consistent with surface-accessible topology. Averages with SD are shown.

Given that the interpretation of our biotinylation results is dependent upon restriction of the NHS-biotin compound by the OM, we also verified surface localization using an antibody-based whole cell assay. We engineered seven different constructs expressing His_8_-ClsO with a single FLAG-tag inserted within the protein sequence. Wild-type 19606 expressing individual FLAG insertion mutants were subjected to a dot blot assay (Fig. 5*A*). Intact cells containing only empty vector or His_8_-ClsO lacking a FLAG insertion show no detection whatsoever. FLAG-tags inserted next to amino acids S21, L44, Q243, and P301 that are predicted to reside along the hydrophilic face of the enzyme (Fig. 5*A*) where easily detected. In comparison, a much lower signal was observed for FLAG insertions at residues K340, G394, and K473. This reduction in signal could be due to lower protein expression, or because the tag location prevents antibody detection. Cell lysates for all constructs were subjected to western blot using both anti-FLAG and anti-His antibodies. A close examination of the western blots indicated the reduced detection of some FLAG insertion mutants could not be solely explained by protein levels. For example, P301 is easily detected on intact cells but anti-His shows similar levels of expression compared to K340, G394, and K473 insertion mutants. Also, K473 shows robust anti-FLAG detection by western blot but not in whole cells.

### Quantitative Analysis Reveals Predominant Surface Topology of ClsO.

Although our initial biotinylation and FLAG epitope mapping experiments support surface exposure of ClsO, these approaches do not resolve whether the enzyme is always at the surface or if only a subpopulation adopts this topology. To directly quantify the extent of surface orientation, we adapted our membrane fractionation and labeling assays to compare biotinylation efficiency when cells remained intact and when lysed (Fig. 5*B*).

Cells expressing His_8_-ClsO were split into two groups. One set of intact cells were treated with NHS-[PEG]_12_-biotin under nonpermeabilizing conditions (whole-cell sample), selectively labeling primary amines exposed on the cell surface. The second set was lysed prior to labeling (pre-lysed control), allowing unrestricted biotinylation of all accessible ClsO molecules, regardless of membrane topology. After labeling, OM fractions were isolated by sucrose gradient fractionation, and levels of total ClsO (anti-His signal) and biotinylated ClsO (streptavidin signal) were measured by dual-channel western blot.

To estimate the fraction of surface-exposed ClsO, we first calculated the ratio of His to biotin signal (His/biotin) within each condition. This within-sample ratio reflects the fraction of total ClsO accessible to biotinylation, while normalizing for differences in sample recovery or protein loading. In the pre-lysed controls, the ratio reflects maximal labeling capacity, as both periplasmic- and surface-exposed ClsO will be accessible. In contrast, the ratio from whole-cell samples reflects only the surface-exposed population. Comparing the ratios between the two conditions provides a relative measure of topological distribution: a His/biotin ratio in whole cells that closely matches the pre-lysed ratio suggests the majority of ClsO is surface-exposed. Across two biological replicates with technical triplicates, the His/biotin ratio in whole cells was ~0.79 of the pre-lysed control (Fig. 5*B*), indicating that ~80% of the available ClsO is accessible to surface biotinylation, consistent with the majority adopting an outward-facing orientation in the OM.

With the exception of its lipid anchor, ClsO is predicted to be a soluble protein. Both the selective-surface biotinylation data and the random FLAG-insertion screen support a model in which the entire protein is surface-exposed. Further, the majority of the enzyme adopts this topology permitting ClsO to access mislocalized GPLs in the outer leaflet and catalyze CL formation directly in the compartment where LOS also resides.

### Disruption of OM Asymmetry in *A. baumannii* Increases ClsO Activity.

**Fig. 6.**
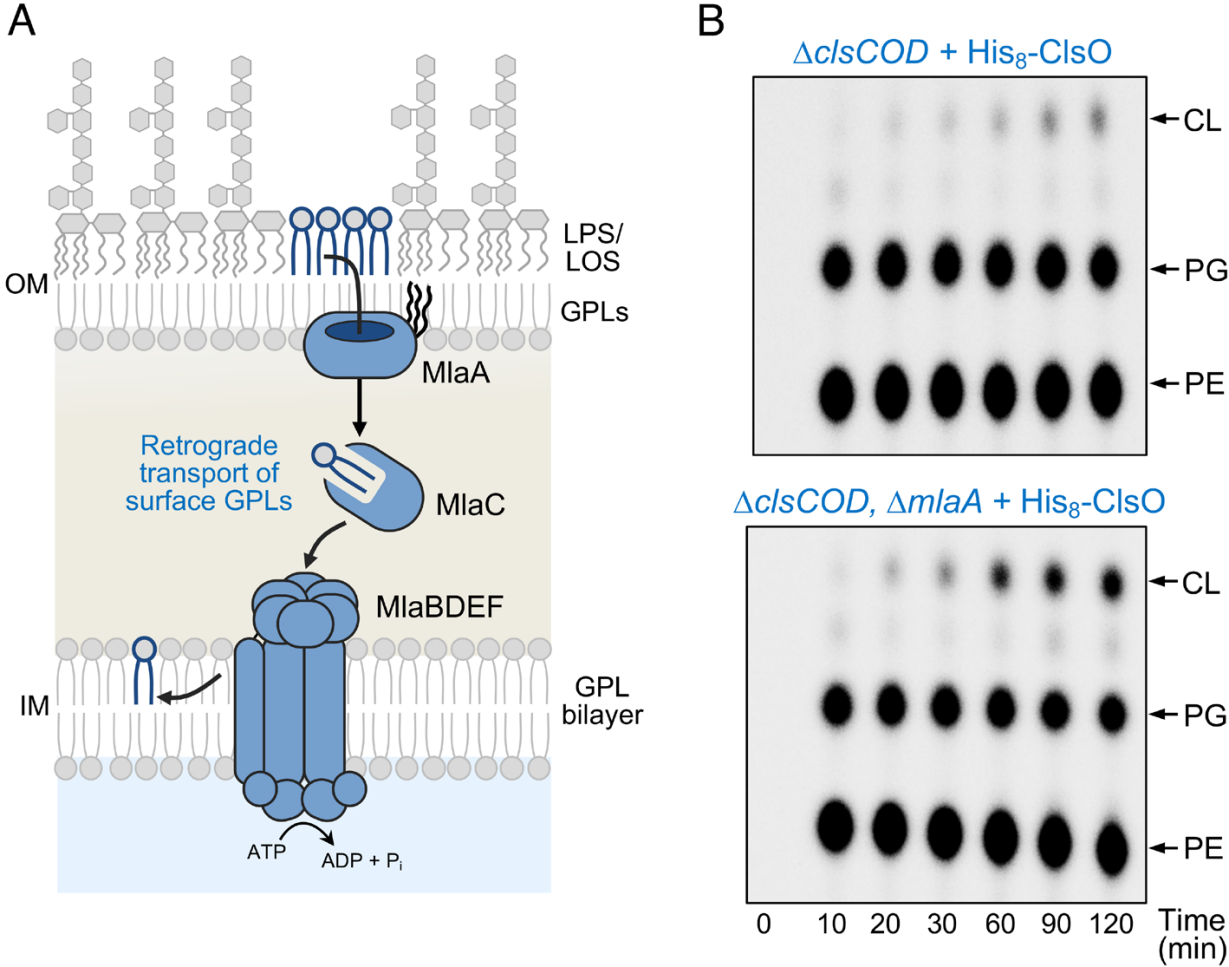
Disruption of OM asymmetry increases ClsO activity. (*A*) Removal of mislocalized GPLs, (e.g., PE, PG) in the outer leaflet of the OM by the Mla retrograde lipid transport system. After translocation across the OM by MlaA, intact GPLs are carried across the periplasm by the lipid chaperone MlaC. The lipid is then inserted into the IM in an ATP-dependent process by the MlaBDEF complex. Schematic depicting Mla function is modeled after that reported by Guest et al. (46). (*B*) The level of ClsO-dependent CL synthesis was compared in *A. baumannii* with and without a functional Mla system (Δ*mlaA* strain). ClsO expression was induced with 50 μM IPTG at an OD_600_ of ~0.2. At OD_600_ of ~0.5, ^32^P_i_ was added and cells were allowed to grow for the indicated times, the GPLs extracted and analyzed by TLC. Loss of Mla-dependent retrograde transport resulted in increased CL synthesis. Data are representative of a minimum of three biological replicates.

Since our data predicts that ClsO synthesizes CL directly at the cell surface, we examined how the availability of GPL substrates in the OM outer leaflet impacts enzymatic activity. Under normal conditions, GPLs that mislocalize to the outer leaflet of the OM are removed by the maintenance of lipid asymmetry (Mla) pathway (Fig. 6*A*) (43, 44). The Mla system, MlaABCDEF, functions as an ATP-dependent retrograde transport pathway that shuttles GPLs back to the IM and is considered the dominant mechanism for maintaining OM asymmetry. Given the Mla system acts to limit GPL accumulation in the outer leaflet, we reasoned if ClsO functions at the cell surface, its activity should increase when this system is compromised. Using our CL-deficient *Acinetobacter* strain expressing ClsO on a plasmid, we deleted *mlaA*. MlaA is responsible for the first step of retrograde transport and is responsible for the selective translocation of outer leaflet GPLs for their eventual reinsertion into the IM (Fig. 6*A*) (45). After induction of ClsO, cells were pulse-labeled with ^32^P_i_ and changes in GPL composition monitored over time (Fig. 6*B*). Notably, relative to the parental strain, deletion of *mlaA* led to a substantial increase in CL production during pulse-labeling supporting the notion the active site of ClsO is on the extracellular face of the OM.

### OM Localization Is Required for ClsO Activity.

**Fig. 7.**
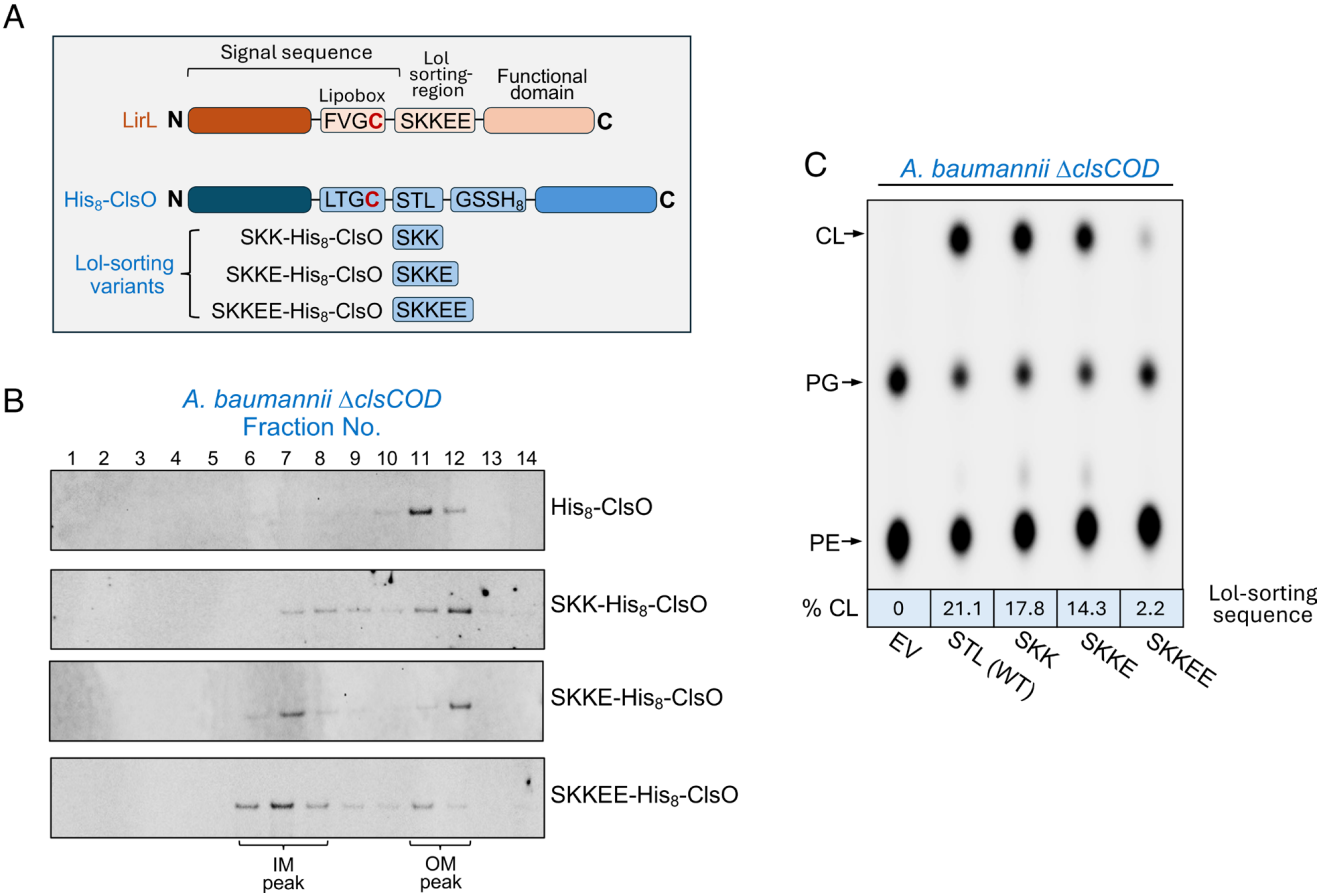
Changing the Lol sorting signal redirects ClsO to the IM and results in reduced CL synthase activity in *A. baumannii*. (*A*) Lipoprotein signal sequence and the predicted Lol sorting region of LirL, ClsO, and ClsO Lol-sorting variants are shown. LirL was previously shown to be localized to the IM of *A. baumannii* and its Lol-sorting region used as a guide for alteration of ClsO. (*B*) Membrane separations of CL-deficient *A. baumannii* expressing His-tagged ClsO Lol-sorting variants. IM and OM peak fractions were determined as described in Fig. 3 and are indicated. Alteration of the ClsO sorting signal to resemble that of LirL resulted in retention of the enzyme in the IM fraction. (*C*) Enzymatic function of each ClsO sorting variant was assessed for CL production in whole cells. As localization of ClsO increased to the IM fraction, less activity was observed. Data in *B* and *C* are representative of three biological replicates.

The vast majority of Gram-negative lipoproteins reside in the OM and are transported to their final destination by the Lol pathway (47). Whether or not a lipoprotein engages with the Lol transport system is determined by the amino acid sequence (Lol-sorting region) following the invariant lipidated cysteine, Cys^+1^, that becomes the N-terminal amino acid after cleavage of the signal peptide (Fig. 7*A*). The rules that govern Lol engagement have been investigated for only a few species and vary (47–49). In *E. coli*, for example, both the +2 and +3 residues influence Lol sorting whereas in *Pseudomonas aeruginosa* Lol engagement appears to be determined by the +3 and +4 residues (50, 51). The rules of lipoprotein sorting in *A. baumannii*, however, remain unknown.

We were curious if we could alter the membrane localization of ClsO and whether localization would impact enzymatic activity. To do this, we altered the amino acid sequence of the ClsO Lol-sorting region to resemble that of LirL (prolipoprotein signal peptidase inhibitor resistance lipoprotein) (Fig. 7*A*), a previously characterized *A. baumannii* lipoprotein that resides primarily in the IM (52). For the first His_8_-ClsO variant, we modified the native +2 to +4 residues of ClsO to match those of LirL (SKK) and observed an increase in IM-localized ClsO (Fig. 7*B*). Variants with additional mutations in the +5 (SKKE-His_8_-ClsO) and +6 positions (SKKEE-His_8_-ClsO) further increased ClsO retention in the IM. Interestingly, as the level of IM-localized ClsO increased in the cell, we observed the level of CL production decreased (Fig. 7*C*) indicating that proper OM localization is necessary for activity. Compared to wild type, there was a ~10-fold reduction in CL synthesis in the SKKEE variant.

In *E. coli*, all Lol-sorting variants remained primarily in the OM and restored CL synthesis to comparable levels (*SI Appendix*, Fig. S6), consistent with proper OM localization. This finding is important, as it indicates that reduced activity of these variants in *A. baumannii* stems from mislocalization to the IM, not from alterations in protein expression or gross folding defects. Western blot analysis of whole-cell lysates showed that Lol-variants were expressed at similar or even elevated levels compared to wild-type ClsO (*SI Appendix*, Fig. S6*A*), and all variants remained membrane-associated (*SI Appendix*, Fig. S6*B*), ruling out instability or misprocessing. We also considered the possibility that the reduced activity observed in *A. baumannii* might reflect altered charge interactions or impaired bilayer engagement due to changes in the Lol-sorting region. However, several observations argue against this interpretation. All constructs include an extended linker between the lipidated cysteine and the globular catalytic domain, which likely provides sufficient flexibility to buffer local charge effects. Moreover, if charge interference near the membrane anchor were the dominant cause of inactivity, one would expect changes in enzymatic activity regardless of membrane context. Yet these same variants retain function in *E. coli*, strongly suggesting that ClsO requires the OM environment for efficient catalysis.

### CL Synthesis in the OM Is Important for OM Integrity.

**Fig. 8.**
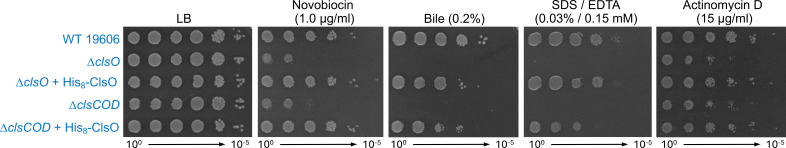
Production of CL at the OM impacts OM integrity. Serial dilutions of the indicated strains were spotted on LB agar or plates containing either novobiocin (1 µg/mL), bile salts (0.2 %), SDS/EDTA (0.03%/0.15 mM), or actinomycin D (15 µg/mL) and grown at 37 °C. Cells lacking *clsO* or all CL synthases were sensitive, and resistance could be restored with only ClsO expression. Data are representative of a minimum of three biological replicates.

To investigate whether CL synthase mutants have any defects in OM integrity, we assayed their sensitivity to novobiocin, an antibiotic that typically does not cross the OM. Deletion of *clsC*, *clsD,* or both (Δ*clsCD*) did not impact growth on LB agar containing 0.5 or 1.0 μg/mL novobiocin, but cells lacking *clsO* were sensitive (Fig. 8 and *SI Appendix*, Fig. S7*A*). Similarly, deletion of *clsO* in Δ*clsC* or Δ*clsD* resulted in novobiocin sensitivity indicating that ClsO-generated CL is critical for the OM permeability barrier (*SI Appendix*, Fig. S7*A*). A decrease in resistance to the large, amphipathic antibiotic actinomycin (15.0 μg/mL) was also observed (Fig. 8), a compound that typically cannot cross the OM barrier. *clsO* mutants showed sensitivity to membrane perturbing agents as well, with a complete loss of growth in agar containing 0.2% bile salts or 0.03% SDS with 0.15 mM EDTA, a chelator of divalent cations that destabilizes LOS/LPS interactions in the OM. In each case, growth could be restored when *clsO* was provided in trans (Fig. 8). Moreover, expression of only ClsO was sufficient to restore growth of the CL-deficient strain (Δ*clsCOD*) on novobiocin and actinomycin D and provided partial rescue when challenged with bile and SDS/EDTA (Fig. 8). Rescue of novobiocin resistance also correlated with ClsO membrane localization and enzymatic activity, as the SKKEE Lol-sorting variant was unable to restore growth in the presence of the antibiotic (*SI Appendix*, Fig. S7*B*).

To ensure the observed changes in antimicrobial resistance were not due to perturbations in LOS structure or its synthesis, we evaluated LOS profiles. Proteinase K-treated whole-cell lysates of wild-type 19606 and each mutant were separated by SDS-PAGE and LOS species stained with Pro-Q Emerald 300 carbohydrate dye. As noted previously (39), wild-type *A. baumannii* produced two major LOS species, one with a fully extended core-oligosaccharide and a second truncated LOS chemotype (*SI Appendix*, Fig. S8*A*). Loss of any one CL synthase or total loss of CL did not impact the LOS banding pattern or LOS levels. The lipid A domain of LOS was also analyzed as changes in the lipid anchor of LOS can greatly alter OM integrity. Again, we saw that alterations in CL synthesis had no impact (*SI Appendix*, Fig. S8*B*).

### ClsO Homologs Are Widely Conserved and Functionally Interchangeable Across Proteobacteria.

**Fig. 9.**
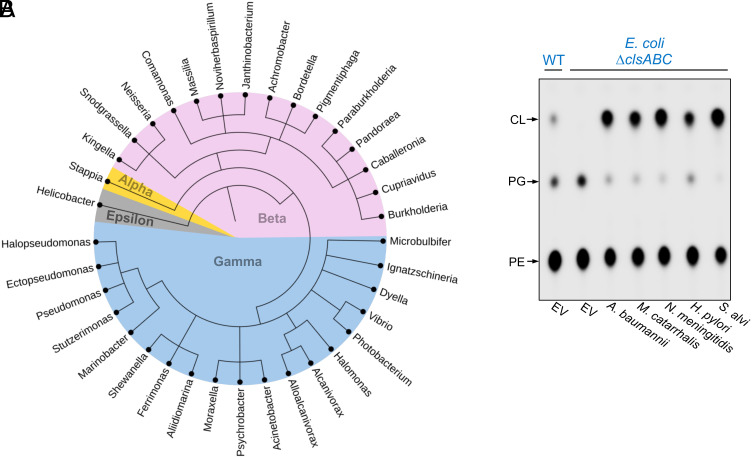
Homologs of ClsO among Proteobacteria. (*A*) Phylogenetic tree of 30 representative genera containing ClsO homologs, see Dataset S1 for complete list of species with ClsO homologs. ClsO homologs were identified via BLASTp and validated by the presence of a lipoprotein signal sequence and two HKD motifs, see *Materials and Methods* for more details. (*B*) Homologs of ClsO from *Moraxella catarrhalis*, *Neisseria meningitidis*, *Helicobacter pylori*, and *Snodgrassella alvi* were expressed in CL-deficient *E. coli* (Δ*clsABC*) under the control of an arabinose inducible promoter (0.02% arabinose). Cells were labeled with ^32^P_i_ and GPLs analyzed by TLC and visualized by phosphorimaging. Data are representative of a minimum of three biological replicates.

To assess the evolutionary conservation of ClsO and evaluate whether surface-exposed CL synthesis might be a broader strategy among Gram-negative bacteria, we performed a comprehensive bioinformatic screen. Using BLASTp analysis and SignalP, we identified ClsO homologs across multiple classes of Proteobacteria, (Fig. 9*A* and Dataset S1). All high-confidence homologs contained predicted lipoprotein signal sequences and retained the characteristic dual HKD motifs, suggesting preservation of enzymatic architecture. The presence of ClsO was not conserved within a particular genus, however. For example, pathogenic species of *Vibrio* such as *Vibrio parahaemolyticus* and *Vibrio vulnificus* encode a ClsO homolog, but it is absent in strains of *Vibrio cholerae*.

We chose representative enzymes from multiple classes of Proteobacteria including *M. catarrhalis*, *N. meningitidis*, *H. pylori*, and *S. alvi* for further analysis. AlphaFold structural models of each show high structural similarity with ClsO, including correct folding of the catalytic core and conservation of membrane-interacting surfaces. To functionally test whether these homologs could synthesize CL, each protein was heterologously expressed in CL-deficient *E. coli* (Δ*clsABC*) and all resulted in robust CL production (Fig. 9*B*). These results suggest that OM-localized CL synthesis is not restricted to *Acinetobacter* but likely represent a conserved mechanism in diverse Gram-negative bacteria.

## Discussion

The cell envelope of Gram-negative bacteria represents a defining and protective feature that allows these organisms to thrive in hostile environments, including the human host. Central to this defense is the asymmetric OM, which restricts the entry of antimicrobial compounds and other environmental stressors. This barrier function relies on the enrichment of LPS or LOS in the outer leaflet and GPLs in the inner leaflet of the OM. Yet, in this study, we identify and characterize ClsO, a surface-exposed OM lipoprotein that synthesizes CL in *A. baumannii*. This finding challenges the longstanding model that bacterial GPL biosynthesis is confined to the cytoplasmic compartment of the cell. While GPLs such as PE, PG, and CL have traditionally been viewed as products of IM-associated enzymes, our results show that CL can also be synthesized at the bacterial cell surface. Therefore, our findings extend the landscape of bacterial lipid metabolism and membrane biogenesis to include the outer leaflet of the OM.

Multiple lines of evidence support this conclusion. ClsO was found to be a lipoprotein localized to the OM (Fig. 3 and *SI Appendix*, Fig. S5), and surface biotinylation experiments coupled with proteomics demonstrated that the active of the enzyme is surface-exposed. Mapping of biotinylated lysine residues to the predicted AlphaFold structure revealed that these sites all reside on the hydrophilic face of the protein, opposite the predicted membrane-interacting catalytic surface, consistent with extracellular presentation of the active site (Fig. 4). Further, analysis of individual FLAG-tag insertion mutants clearly showed that ClsO could be easily detected on the surface of whole cells (Fig. 5*A*). Although ClsO is also functional in the OM of *E. coli*, its final topology in this heterologous host remains unresolved. Still, quantitative labeling experiments in *A. baumannii* estimated that the vast majority of ClsO (~80%) was accessible to biotinylation (Fig. 5*B*). These findings support a model in which ClsO resides predominantly on the cell surface, positioning its active site to directly access GPLs that spontaneously flip to the OM outer leaflet.

Further mechanistic support for surface activity comes from functional studies showing that OM localization of ClsO is essential for function (Fig. 7 and *SI Appendix*, Fig. S6). When the Lol-sorting region of ClsO was mutated to mimic that of a known IM lipoprotein, the enzyme was redirected to the IM and its ability to generate CL was markedly reduced. This loss of activity was not due to instability or misfolding, as protein levels were comparable to those of native ClsO. Additionally, deletion of *mlaA*, a core component of the Mla system responsible for retrograde GPL transport from the OM to the IM, significantly increased ClsO activity (Fig. 6). This result is consistent with a model in which mislocalized GPLs accumulate in the outer leaflet in the absence of Mla and are utilized as substrates by surface-exposed ClsO. Taken together with prior evidence showing the dynamics of GPL flipping and Mla-mediated retrograde repair (25, 43–45, 53), these results illustrate that even under nonstress conditions a small but continuous flux of GPLs reaches the OM outer leaflet. ClsO, when present on the surface, can directly compete for this mislocalized GPL pool, and overexpression of ClsO can readily outpace Mla activity, leading to robust CL production.

The biological significance of surface CL synthesis is underscored by its impact on membrane integrity. Cells lacking *clsO*, but not *clsC* or *clsD*, were sensitized to detergents, bile salts, and key antibiotics (Fig. 8 and *SI Appendix*, Fig. S7). These phenotypes were not due to alterations in LOS production or changes in LOS/lipid A structure (*SI Appendix*, Fig. S8), suggesting that the defects reflect compromised OM barrier function. Notably, OM-localized ClsO, but not IM-retained variants, could restore resistance, reinforcing that ClsO function at the cell surface is critical for physiological resilience. One possible explanation for this phenotype is that surface-localized CL engages in divalent cation cross-bridging, much like LOS or LPS. Unlike PE and PG, CL carries two phosphate headgroups and thus has a high anionic charge density that facilitates Ca^2+^ or Mg^2+^-dependent cross-linking between lipids, enhancing membrane stability. In this model, CL would act in a manner analogous to LOS, helping to rigidify the OM and reduce permeability to hydrophobic molecules.

Importantly, the production of CL in the outer leaflet may also serve as a mechanism to remove excess PE and PG from this compartment. Given ClsO is most like ClsC, the condensation reaction catalyzed by the enzyme consumes one molecule each of PG and PE, both of which are aberrant in the outer leaflet. Thus, ClsO could function in a manner similar to the Mla system, not simply generating CL but removing detrimental lipids. This enzymatic strategy may be particularly useful under stress conditions where retrograde transport is overwhelmed or insufficient. Rather than returning misplaced GPLs to the IM, ClsO offers an additional route: converting them into a stabilizing lipid with unique biophysical properties at the membrane surface.

Our data also reveal important differences in lipoprotein sorting signals between *A. baumannii* and *E. coli*. While the Lol-sorting region in *E. coli* typically relies on residues at the +2 and +3 positions following the lipidated cysteine, our experiments showed that equivalent changes in ClsO sequence did not redirect it in *E. coli*, whereas similar mutations profoundly altered localization in *A. baumannii*. This highlights that the rules governing lipoprotein trafficking are species-specific and remain poorly understood in many clinically relevant pathogens. Related studies in *Pseudomonas* (50, 51) have shown genus-specific sorting cues that also diverge from *E. coli.* Our findings reinforce the need to define Lol-sorting determinants across a wider range of Gram-negative bacteria.

Comparative genomics revealed that ClsO homologs are broadly distributed across multiple classes of Proteobacteria, including α-, β-, γ-, and ε-Proteobacteria. All identified homologs retained conserved HKD catalytic motifs and lipoprotein signal peptides. When expressed in a CL-deficient *E. coli* strain, ClsO homologs from diverse species (*Neisseria*, *Moraxella*, *Snodgrassella,* and *Helicobacter*) restored CL synthesis, confirming their functionality. Collectively, the data argue that OM-localized CL synthesis is not a rare adaptation of *A. baumannii*, but rather a conserved and perhaps ancient feature of Gram-negative envelope biology.

Considering these findings, it is worth acknowledging prior studies that independently knocked out the *clsO* gene in both *A. baumannii* (54) and *Neisseria gonorrhoeae* (55). In both cases, the authors proposed that the protein was a secreted lipase that damaged host cell membranes, an interpretation that stemmed from sequence homology to phospholipase D enzymes. Despite this misannotation, both studies found that *clsO* mutants exhibited reduced fitness in mammalian infection models, reinforcing the idea that ClsO plays a crucial and conserved role in host-associated survival.

In the future, it will be important to explore the broader functional consequence of CL production at the bacterial surface. CL plays multifaceted roles in bacterial physiology, including modulating membrane structure, influencing protein localization, and enhancing enzymatic activity. The lipid clusters into membrane microdomains, especially at cell poles and division septa, where its conical shape and high anionic charge promote membrane curvature and spatial organization (14, 56). CL also serves as a lipid landmark for recruiting or stabilizing membrane proteins and can stimulate the activity of membrane-complexes. For instance, CL promotes SecYEG-mediated protein translocation (57, 58) and CL supports MsbA-mediated LPS transport (13). These findings suggest ClsO-mediated CL synthesis could influence not just OM integrity, but also the organization of membrane domains and the function of OM proteins.

Altogether, the work presented here reveals that CL synthesis can occur at the bacterial cell surface via an OM-localized, surface-exposed lipoprotein. This finding challenges a longstanding assumption in bacterial cell biology and expands our understanding of envelope biogenesis. By extending GPL synthesis beyond the IM, bacteria may exert spatial control over membrane composition, potentially tuning envelope properties in response to stress or environmental cues. The broad conservation of ClsO homologs among Gram-negative species suggests that this is not a rare adaptation, but a conserved strategy for modulating OM architecture. As the OM serves as a critical barrier to antibiotic entry, uncovering how lipid distribution and homeostasis are maintained at this interface may provide additional targets for disrupting bacterial defenses.

## Materials and Methods

### Strains, Plasmids, and Bacterial Growth.

All strains and plasmids used in this study are listed in Dataset S1. All *A. baumannii* and *E. coli* strains were grown in LB broth or on LB agar plates incubated at 37 °C. For selection of strains containing plasmids, 100 μg/mL of ampicillin, 30 µg/mL kanamycin, 7.5 µg/mL tetracycline, 30 µg/mL tellurite, 60 µg/mL apramycin (*E. coli*), or 200 µg/mL apramycin (*A. baumannii*) were used when appropriate. The remaining methods are provided in *SI Appendix*, including recombinant DNA techniques, generation of mutants, lipid A analysis, experiments to determine cell envelope localization, etc.

## Supplementary Material

Appendix 01 (PDF)

Dataset S01 (XLSX)

## Data Availability

All data related to this paper are included within the Zenodo research data repository (https://doi.org/10.5281/zenodo.17651785) (59). All other data are included in the manuscript and/or supporting information.
