# Supplementary material for: A surface-exposed cardiolipin synthase provides an unexpected paradigm for maintaining the Gram-negative outer membrane
Source: Proc Natl Acad Sci U S A. 2026 Jan 22;123(4):e2524588123. doi: 10.1073/pnas.2524588123 (PMC12846801; doi:10.1073/pnas.2524588123)
Supplement: Supplementary file 1 — Appendix 01 (PDF) [file pnas.2524588123.sapp.pdf]

SUPPORTING INFORMATION: A surface-exposed cardiolipin synthase provides a new paradigm in maintaining the Gram-negative outer membrane (Herrera and co-workers).

This file includes the following:

Supporting text
Figures S1-S8
Tables S1-S4
SI References

Other supporting materials for this manuscript include the following:

Datasets S1
Other data can be found on Zenodo research data repository (10.5281/zenodo.17651785)

Supplemental Methods

Recombinant DNA techniques—Custom synthetic oligonucleotides listed in **Dataset S1** were synthesized by Eurofin Genomic. PCR products were obtained using Takara Ex-Taq DNA polymerase (Takara), resolved in 1% agarose gel and purified using the Quiaquick gel extraction kit (QIAGEN). DNA was cleaned and concentrated with the DNA clean and concentrator kit (Zymo Research). Apex^{TM} Taq RED polymerase was used for colony PCR screening of vector constructs or deletion mutants. Genomic DNA extraction was performed using Easy-DNA genomic DNA (gDNA) purification kit (Invitrogen). Plasmid DNAs were extracted using QIAprep spin miniprep kit (Qiagen). To create recombinant plasmids all restriction enzymes, T4 DNA ligase and Antarctic phosphatase were purchased from New England Biolabs (NEB). Mutations in a specific gene within a plasmid were introduced using the Q5 Site-Directed mutagenesis kit (NEB). All recombinant vectors were subjected to whole-plasmid sequencing to confirm sequence accuracy.

Chromosomal mutagenesis—Generation of *cls* mutants in strain 19606: 19606 *cls* gene sequences annotated as *HMPREF0010_00607* (*clsC*), *HMPREF0010_03706* (*clsO*), and *HMPREF0010_02731* (*clsD*) were obtained from Biocyc Genome Database Collection (1).
Single deletion mutants of *clsC* and *clsD* in strain 19606 were obtained by RecET-mediated recombineering as previously described (3). Briefly, pKD4 plasmid (4) was used as the DNA template for PCR amplification of *clsC*::km and *clsD*::km using 19 P1-P2 Δ*clsC* and 19 P1-P2 Δ*clsD* primers, respectively (**Dataset S1**). 19606 carrying pMM-REC$_{Ab}$ plasmid for homologous recombination was transformed with PCR products and allele deletions were verified by PCR using ver-F/ver-R primers corresponding to each deletion (**Dataset S1**). Plasmid pMM-REC$_{Ab}$ from mutant strains was cured after several growing cycles without selection agent. To generate marker-less *clsC* and *clsD* mutants, cells were transformed with pMM-FLP plasmid (**Dataset S1**). Kanamycin resistance cassette excision was verified by PCR and subsequently the pMM-FLP plasmid was cured.

We used scar-free genome editing protocol (5) for in frame *clsO* deletion, because RecET-mediated recombineering system was unsuccessful. Briefly, a 986 bp upstream homologous region that included the first 27 bp of the 5′-end of the *clsO* open reading frame was PCR amplified using primers upAbclsO-BamHI and upAbclsO-R (**Dataset S1**). The downstream homologous region containing 27 bp of the 5′-end complementary sequence, the last 180 bp at 3′-end, and the 828 bp downstream of *clsO* was obtained using primers downAbclsO-F and downAbclsO-R (**Dataset S1**). The PCR product of the downstream region contains an internal EcoRI restriction

site near to the 3′-end that was used for subsequent cloning, and the 27-bp at 5′-end complementary sequence served as an overlapping region. Both upstream and downstream fragments were spliced together by SOEing PCR (6). The resulting PCR product was EcoRI-BamHI double digested and cloned into the multiple cloning site (MCS) of the suicide plasmid pEMGT (**Dataset S1**), yielding the plasmid pEMGT::Δ*clsO*.

The pEMGT::Δ*clsO* construct was introduced into SM10 λ*pir* and transformants were selected with tellurite. The resulting strain was used as the donor to transfer the vector into wild-type 19606 by biparental mating and candidate transconjugants containing the integrated plasmid were selected with tellurite and chloramphenicol. Importantly, the integrated pEMGT plasmid backbone contains SceI target sites, 18-bp in length, that flank the MCS. To promote the second homologous recombination event and pEMGT elimination, plasmid pSW-apramycin was introduced into 19606-pEMGT::Δ*clsO* by electroporation followed by plating on apramycin. pSW-apramycin (**Dataset S1**) encodes the endonuclease SceI that introduces a double-strand break in the chromosome inducing DNA repair and pEMGT elimination. Colonies that grew on plates containing apramycin, but not apramycin and tellurite were considered positive for *clsO* deletion. Deletions were confirmed by PCR using primers verclsO F/R (**Dataset S1**). At this step, pSW-apramycin was cured from the scar-free Δ*clsO* strain.

To generate the Δ*clsCD* strain, Δ*clsC* was subjected to a second round of recombineering targeting *clsD* as noted above. To create Δ*clsCO* and Δ*clsDO* strains, the *clsC* and *clsD* single mutants and SM10 λ*pir* pEMGT::Δ*clsO* strains were mated. Selection of transconjugants and subsequent steps followed the scar-free editing protocol described above. The CL-deficient *cls* triple mutant (Δ*clsCOD*) was generated using Δ*clsCO* and performing an additional round of recombineering targeting *clsD*. Finally, deletion of *mlaA* in Δ*clsCOD* were generated by RecET-mediated recombineering using the *mlaA*::km (**Dataset S1**) PCR products.

<u>*cls* mutants in strain 5075</u>: AB5075 parental strain and derived mutants *ABUW_RS10015* (*clsC*), *ABUW_RS02470* (*clsO*) and *ABUW_RS02955* (*clsD*) were obtained from the *A. baumannii* AB5075 ordered Tn mutant library (2).

Generation of plasmid constructs—All ORFs were obtained from Biocyc database (1). The *clsO*$_{Ab}$ and C-terminal *clsO*$_{Ab}$-His$_8$ gene sequences were codon optimized, synthesized and cloned into pET21a+ vector by Genscript (**Dataset S1**). These constructs served as template for further subcloning. Plasmids pBAD18 and pMMB67EH were used for gene expression in *E. coli* whereas pMMB67EH was used in *A. baumannii*. *clsO*$_{Ab}$ and *clsO*$_{His8\text{-}Ab}$ were subcloned into pBAD18 using KpnI-HindIII restriction sites and primers pB1 F/R and pB1 F/pB2 R generating pClsO$_{Ab}$ and pClsO-His$_{8\text{-}Ab,}$ respectively. N-terminal His$_8$-tagged ClsO was carried out by site directed mutagenesis using primers pBN3-F/R and pClsO$_{Ab}$ template yielding pHis$_8$-ClsO$_{Ab}$ (**Dataset S1**). Since the N-terminal His-tagged ClsO$_{Ab}$ variant maintains full enzymatic activity, it was used for all further experimentation.

The STL$^{(+2 \text{ to } +4)}$ Lol-sorting region of ClsO$_{Ab}$ was converted to the putative Lol-sorting region of LirL$_{Ab}$ substituting the +3 threonine and +4 leucine residues with lysines using primers pK2 F/R and pMMHis$_8$ClsO$_{Ab}$ as template. Similar mutations were made to generate the ClsO variants KKE (primers pK2E F/R) and KKEE (primers pK2E2 F/R) (**Dataset S1**). ClsO$_{Ab}$-1x FLAG variants were engineered inserting the sequence encoding the epitope DYKDDDDK by site-directed mutagenesis (Genscript) in pMMHis$_8$ClsO$_{Ab}$ plasmid. FLAG variants were named based on the amino acid and position preceding the tag insertion in the mature protein (**Dataset S1**).

The *A. baumannii mlaC* gene was cloned into double digested EcoRI/BamHI pMMB67EH using primers pMlaC F/R yielding the plasmid pMM-MlaC$_{Ab}$ (**Dataset S1**). This construct served as the template for subsequent site-directed mutagenesis to insert an N-terminal His-tag after the signal peptide using primers pHMlaC F/R (**Dataset S1**)

Analysis of ^{32}P-labeled GPLs—Cells were grown in LB at 37°C in the presence of $^{32}P_i$ (2.5 or 5.0 μCi/ml for *E. coli* and *A. baumannii,* respectively) to an OD_{600} of ~1.0. Cells were harvested by centrifugation and the GPLs isolated by Bligh-Dyer extraction as previously described (7, 8). GPLs (~20,000 cpm/sample) were separated by TLC using a chloroform/methanol/acetic acid (65:25:10, v/v/v) solvent system. Lipids were visualized by phosphorimaging analysis using Amersham Typhoon Biomolecular Imager (Cytiva) and changes in GPL production were quantified by densitometry. Images shown have been adjusted to allow visibility of all lipid species; however, an unsaturated image was used for all densitometry calculations.

Efficiency of Plating Assays—Overnight cultures were adjusted to the same OD_{600} of 1.0 and serially diluted in LB broth in a 96-well microtiter plate. Dilutions were spotted onto LB agar or agar containing the specified antibiotic, 0.2% bile, or SDS/EDTA (0.03%/0.15mM) along with 50 μM IPTG. Bacteria were grown overnight at 37°C.

Isolation and analysis of ^{32}P-labeled lipid A—For lipid A analysis, cells were grown in the presence of $^{32}P_i$ (5.0 μCi/ml) to an OD_{600} of ~1.0 and the lipid A isolated as previously described (9, 10). In brief, lipid A was released from the core oligosaccharide via a mild acid hydrolysis (pH 4.5, 100°C) and isolated via Bligh-Dyer extraction. Lipids (~5,000 cpm/sample) were separated by TLC using a chloroform/pyridine/88% formic acid/water (50:50:16:5, v/v/v/v) solvent system and visualized by phosphorimaging analysis.

Biotinylation assay—Overnight cultures of *A. baumannii* were diluted to OD_{600} 0.05 in LB broth containing antibiotics and protein expression induced at OD_{600} of ~0.2. Cells were harvested at OD_{600} of ~0.6 by centrifugation (5,000 x *g* 10 min). Cell pellets were washed three times in 1X cold PBS and whole cells were resuspended in 1X PBS to reach an OD_{600} of ~6.0 per 0.9 ml. Intact whole cells were labeled with 0.2 mM EZ-link NHS-[PEG]$_{12}$-biotin (Life Technologies) dissolved in 1X PBS, in a final 1 ml reaction volume. Whole cells were biotinylated for 2 min at room temperature and the reaction quenched by the addition of 250 mM Tris HCl pH 7.5. Cells were then vortexed and incubated at room temperature for 10 min. Biotinylated cells were harvested at 10,000 x *g* for 10 min at 4ºC and the resulting pellet washed 3X in cold 1X PBS.

The biotinylated whole cells were then resuspended in 2 ml of 1X PBS buffer containing cOmplete Mini EDTA-protease inhibitor and 50 µg/ml DNAseI. Cells were lysed using French press at 18,000 psi and cell debris removed by centrifugation at 10,000 x *g* for 10min at 4ºC. The resulting cell-free extract was centrifuged at ~200,000 x *g* for 30 min at 4ºC using a Type 50.4 Ti rotor (Optima XE-90 ultracentrifuge, Beckman Coulter). The soluble fraction was collected, and the membrane fraction was washed two times in cold 1X PBS. The final membrane pellet was resuspended in buffer 1X PBS 1% SDS. For labeling of cell lysate, cell pellets were resuspended in cold 1X PBS containing cOmplete Mini EDTA-protease inhibitor and 50 µg/ml DNAseI and lysed via French press at 18000 psi. A volume of 0.9 ml crude extract was labeled with 0.2 mM EZ-link NHS-[PEG]$_{12}$-biotin as outlined above.

Purification of biotinylated proteins was performed as previously described (11) with slight modifications. Briefly, washed labeled whole cell pellets were lysed in buffer of 10 mM Tris-HCl, 250 mM NaCl, 25 mM Imidazole and 1% Triton X-100 for membrane proteins and 0.2% Triton X-100 for soluble proteins. Cell-free extracts were incubated on a rotator for 16h at 4ºC. Solubilized samples were mixed with HisPur Ni-NTA Resin (Thermo Scientific) and rotation continued for 2 h at 4ºC. Purified proteins were eluted using 500 mM imidazole. TCA precipitation was used to concentrate protein samples.

Proteomic mass spectrometry of biotinylated ClsO—Samples were analyzed by MS to identify sites of surface biotinylated of ClsO. Purified biotinylated and non-biotinylated His-ClsO (control) were subjected to SDS-PAGE using a 10% Bis-Tris gel and ran approximately 2 cm into the gel. The gel was stained with colloidal Coomassie blue and the protein bands excised for MS analysis. Protein samples were processed and analyzed by the Georgia Tech Mass Spectrometry Core-Proteomic facility.

Separation of IM and OM fractions—Isopycnic sucrose gradient fractionation was used to separate membranes of both *E. coli* and *A. baumannii*. *E. coli* separations were carried out as using a well-established method (7, 12). As noted in the literature, prior methods used for the separation of membranes from *E. coli* or *Salmonella enterica* are ineffective for *Acinetobacter* (8, 13). For A. baumannii, modifications to the protocol were applied. Briefly, overnight cultures were diluted an OD_{600} of ~0.05 in 30 ml LB broth containing antibiotic. Where needed, 50 µM IPTG was added at an OD_{600} of ~0.2 to induce protein synthesis. Cells were harvested at an OD_{600} ~0.5 and cell pellets washed once in cold 10 mM Tris-HCl pH 8.0. Pellets were resuspended in 6 ml buffer containing 10 mM Tris-HCl pH 8.0, 20% sucrose (w/v), cOmplete mini EDTA-free protease inhibitor and 50 µg/ml DNAseI. The cell suspension was lysed via French press, one passage at 8,000 psi, and debris removed by centrifugation for 4,500 x *g* for 10 min. Next, 5.5 ml of the cell-free extract was layered onto a sucrose gradient consisting of 2.4 ml 45% sucrose (w/v), 2.4 ml 53% sucrose (w/v) and 2 ml 73% sucrose (w/v) in 10 mM buffer Tris-pH 8.0. To separate membrane fractions, samples were ultracentrifuged at 220,000 x *g* at 4ºC for 20 h using SW 41 Ti Rotor (Optima XE-90 ultracentrifuge, Beckman Coulter). At the end of the sucrose gradient sedimentation, 0.8 ml fractions were extracted from the top to the bottom of the tube and collected in 1.5 ml microcentrifuge tubes for further processing.

Analysis of membrane markers—After separation of membranes by sucrose density gradients, fractions across the entire gradient were tested for the presence of NADH oxidase (IM marker) and for the presence of OmpA and LOS/LPS (OM markers). NADH oxidase was determined using a well-defined enzymatic assay (7, 14) using 2.5 µl of either *A. baumannii* or *E. coli* membrane fractions. The level of NADH oxidase activity for each fraction is expressed as a % of total oxidase activity across the entire gradient. For OmpA detection, 10 µl of each fraction was subjected to SDS-PAGE using a 10% Bis-Tris gel followed by detection of OmpA by Western blot (see Western blotting analysis for details) as previously described (7, 8). Finally, the presence of either LOS (*A. baumannii*) or LPS (*E. coli*) in each fraction was assessed. 35.5 µl of each gradient fraction was combined with SDS-PAGE loading buffer containing 5% beta-mercaptoethanol to a final volume of 50 µl and the sample boiled for 10 min. After cooling, 1.5 U of Proteinase K (New England Biolabs) was added and the samples incubated overnight at 55ºC. For LPS/LOS detection, 10 µl of sample was resolved by SDS-PAGE as previously described (15) and stained using Pro-Q Emerald 300 dye LPS gel stain kit (Invitrogen).

Western blotting analysis—Protein samples were resolved using 10% Bis-Tris gels, transferred onto 0.2 μm PVDF membranes, and probed with the appropriate primary antibody. For His-tagged proteins, THE™ His Tag Antibody, mAb-Mouse (Genscript) was used at a dilution of 1:10000. Detection of FLAG-tags was carried out using monoclonal anti-FLAG M2, produced in mouse (Sigma) diluted to 1:5000. For detection of OmpA, OmpA IgG-Rabbit (LSBio) diluted 1:80,000 was used for *E. coli* and 1:5000 for *A. baumannii*. The fluorescent secondary antibodies DyLight 800 Goat Antimouse IgG and StarBright blue 700 Goat Anti-rabbit IgG (Biorad) were used at dilution 1:10000. Biotinylated proteins were detected using 1:5000 Streptavidin conjugated to DyLight 550 (Biorad). Labeled target proteins were visualized using ChemiDoc (Biorad).

Dot blot assay—Overnight cultures were diluted to OD_{600} ~ 0.05 in fresh LB medium containing antibiotic and 100 μM IPTG. Cells were grown at 37°C to mid-log and normalized to OD_{600} ~ 1.0. Four microliters of intact cell suspension were spotted onto 0.2 μm nitrocellulose membranes. The presence of the FLAG-tag was detected as indicated for western blot analysis.

Quantification of ClsO topology in the OM—To quantify the orientation of ClsO in the OM, we adapted the surface biotinylation and membrane fractionation assays described above with minor modifications. Cells expressing His_8-ClsO were grown to mid-log phase in LB broth and split into two equal portions. One half was subjected to surface biotinylation under native conditions ("whole-cells" sample), while the other half was lysed prior to biotinylation ("pre-lysed" control).

For surface labeling (whole-cells), intact cells were biotinylated as described in the "biotinylation assay" section. However, for subsequent cell lysis and membrane fraction steps, 10 mM Tris (pH 8.0) was replaced with 1X PBS in all solutions while other buffer components (e.g., sucrose, protease inhibitor, etc.) were maintained. Biotinylated whole cells were lysed via French press at 8,000 psi, debris removed by centrifugation, and the cell-free extract in 20% sucrose (w/v) subjected to membrane fractionation as described in the "separation of IM and OM fractions" section. To determine maximal labeling capacity of ClsO (pre-lysed sample), cells were broken via French press and biotinylated as described. The final cell free extract was adjusted to 20% sucrose (w/v) in PBS and subjected to membrane fractionation.

The quality of IM and OM separation for each sample was confirmed as described under "analysis of membrane markers." For ClsO quantification, equal volumes of peak OM fractions (F11-F13) were pooled and analyzed by multichannel fluorescence imaging on a Bio-Rad ChemiDoc system. Signal intensities for His-tagged ClsO and biotin-labeled ClsO were detected in Dylight 800 and Dylight 550 channels, respectively, and quantified using Bio-Rad Image Lab 6.1 software.

The ratio of His-signal (total ClsO) to biotin-signal (surface-exposed ClsO) was calculated for both intact whole-cells and pre-lysed samples. This within-sample ratio reflects the fraction of total ClsO that is accessible to biotinylation under the specific labeling condition. Since pre-lysed samples represent the maximal labeling capacity of ClsO in both OM membrane leaflets, we used the ratio of intact-to-lysed signal as a proxy for surface accessibility. A whole-cell/pre-lysed ratio of near 1.0 would suggest predominant surface exposure, whereas lower ratios would be expected if a substantial fraction of ClsO were periplasmic-facing but inaccessible in intact cells.

Evaluation of *A. baumannii* LOS—To evaluate gross changes in LOS structure and/or synthesis, cells were grown in LB broth and grown overnight at 37 °C. Cells equivalent to OD_{600} of 1.0 were collected by centrifugation and resuspended in 100 μl of SDS-PAGE buffer. After

boiling for 10 min, the cell lysate was treated with proteinase K and subjected to SDS-PAGE as previously described (15). LOS was stained using Pro-Q Emerald 300 dye as noted above.

Next-generation sequencing—Strains subjected to whole-genome sequencing are indicated in **Dataset S1**. Bacteria were grown overnight in 5 ml of LB and the bacterial pellet from 1 mL of culture was submitted to SeqCenter, LLC for genomic DNA extraction and whole-genome sequencing. The resulting reads were trimmed, locally realigned and any variations mapped using CLC Genomics Workbench from Qiagen. RNA sequencing data for both wild-type *E. coli* K12 (W3110) and *A. baumannii* (19606) was performed on three cultures at an OD_{600} of ~0.7, 2, and 4 representing mid-log, early stationary, and late stationary phases of growth, respectively. RNA extraction, library building, and sequencing were performed by SeqCenter, LLC in biological triplicate. Reads were mapped to the respective published genomes as noted above.

Structural Predictions and identification of protein motifs—The presence or absence of TM domains, signal peptides, and protein motifs were evaluated using DeepTMHMM (https://dtu.biolib.com/DeepTMHMM), Signal P 5.0 or 6.0 (16, 17), and ProSite (18), respectively. Protein structures were predicted using AlphaFold3 (AF3) through the AlphaFold server (version 2025.01.15) (19), and structures were analyzed using UCSF ChimeraX (20). All protein-coding genes were modeled with automatically generated seeds and the default ten recycles. For each protein, AF3 generates five structural models, each with a predicted template-based modeling (pTM) score. The highest-ranking model based on pTM score was selected for subsequent structural homology analyses.

Bioinformatic analysis—ClsO homologs were initially identified via BLAST using an experimentally verified *clsO* sequence (EEX01694.1). *clsO* homologs were then filtered out if they did not contain a lipoprotein signal sequence and contain two HKD motifs. See **Dataset S1** for a comprehensive list of prediction scores for each *clsO* homolog. After the list of *clsO* homologs was compiled, a phylogenetic tree was generated using PhyloT v2 and then annotated with iTOL (21).

Supporting Figures and Tables

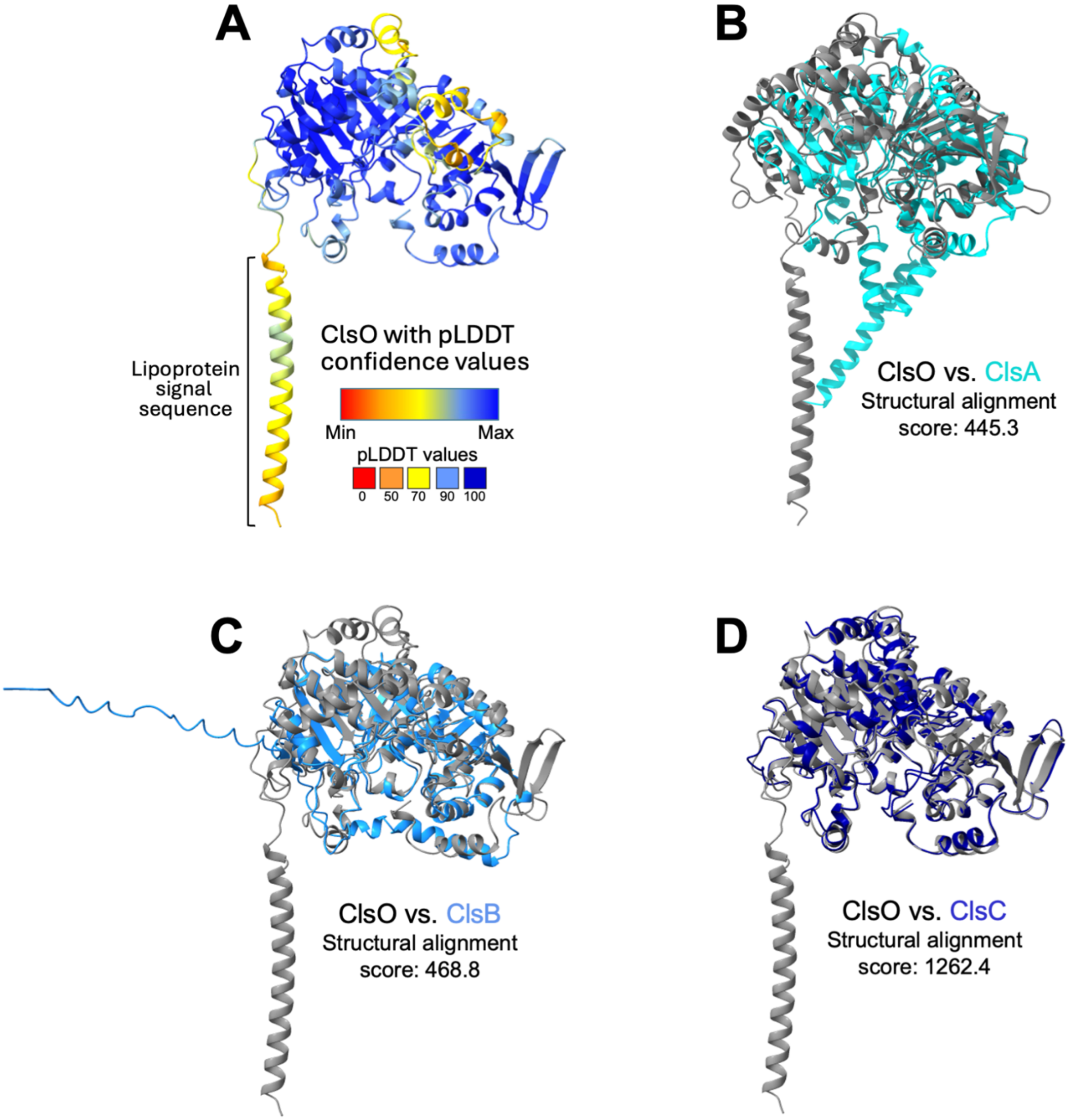

Fig. S1. Comparison of the AlphaFold structure of *A. baumannii* ClsO to *E. coli* ClsA, ClsB, and ClsC. (A) *A. baumannii* ClsO structure with pLDDT confidence for each amino acid (see pLDDT confidence key). The structure also includes the lipoprotein sorting signal. pLDDT values above 90 indicate a high level of confidence. **(B)** Structural comparison of ClsO (grey) vs. *E. coli* ClsA (Cyan). **(C)** Structural comparison of ClsO (grey) vs. *E. coli* ClsB (light blue). **(D)** Structural comparison of ClsO (grey) vs. *E. coli* ClsC (dark blue). Structures were all generated using AlphaFold3 with default prediction parameters. Structural comparisons were all generated using ChimeraX matchmaker. Structural alignment scores were generated using Matchmaker using default settings with ChimeraX.

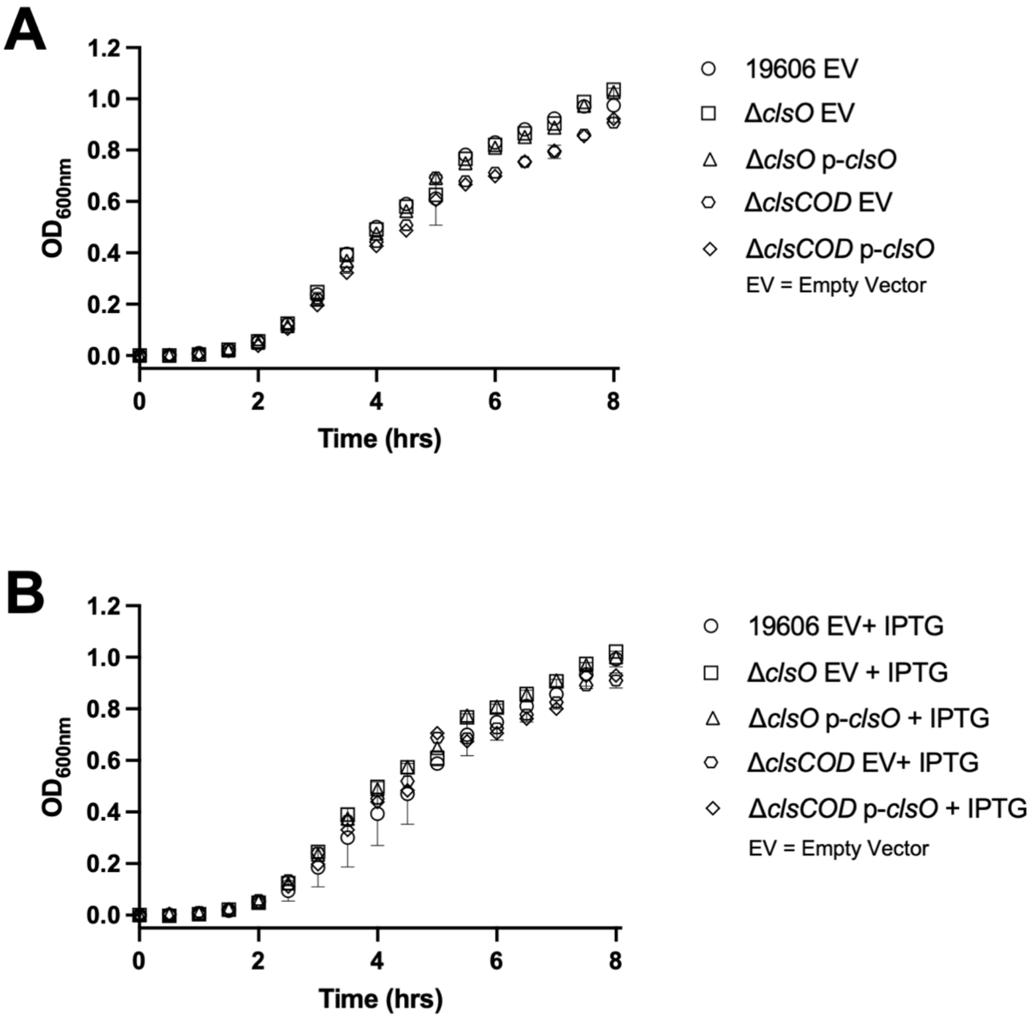

Fig. S2. Growth of *A. baumannii* strains with altered CL production. *A. baumannii* CL synthase mutants and strains overexpressing ClsO (5 ml cultures) were grown in biological triplicate at 37ºC in the absence **(A)** or presence **(B)** of 50µM IPTG. Error bars are not visible if smaller than the symbol.

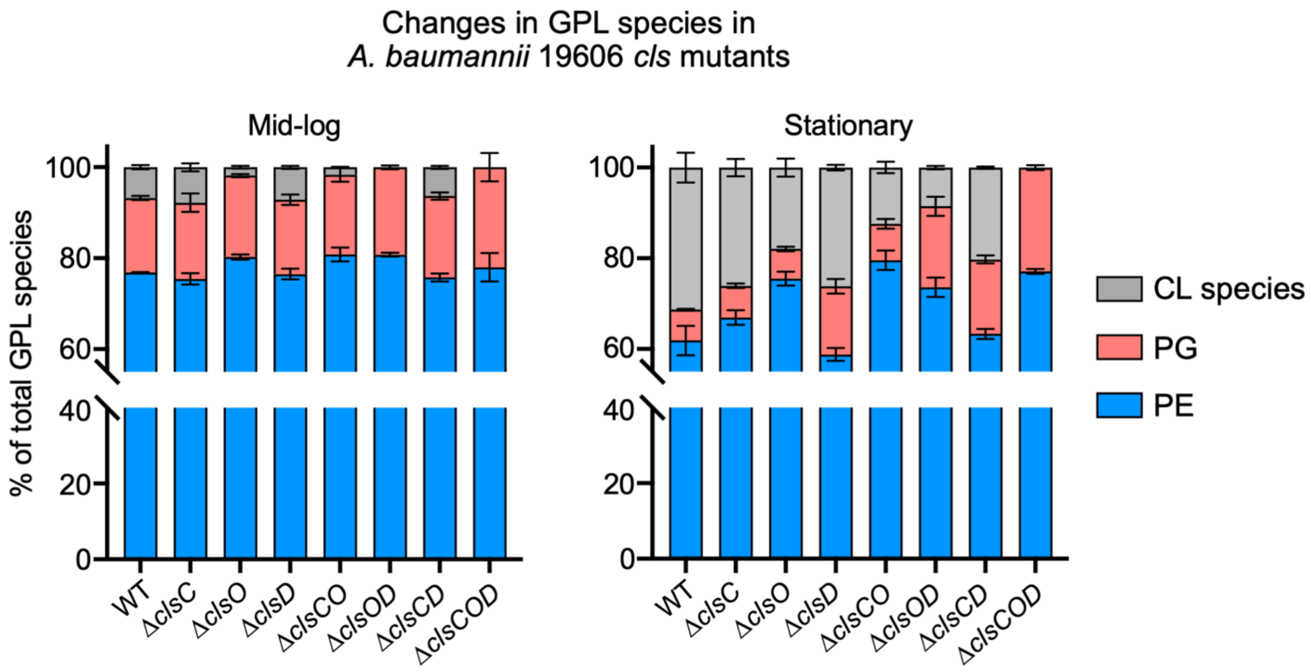

Fig. S3. Analysis of GPL synthesis in *A. baumannii* 19606 *cls* mutants. GPLs from ^{32}P-labeled single, double, and triple *cls* mutants were isolated, separated by TLC, and quantified by phosphorimaging analysis. Quantification of GPL species in mid-log and stationary phase cultures is shown. In both single and double mutants, loss of ClsO results in the largest decrease of CL species and deletion of all three synthases results in no CL production. Data is representative of a minimum of three biological replicates.

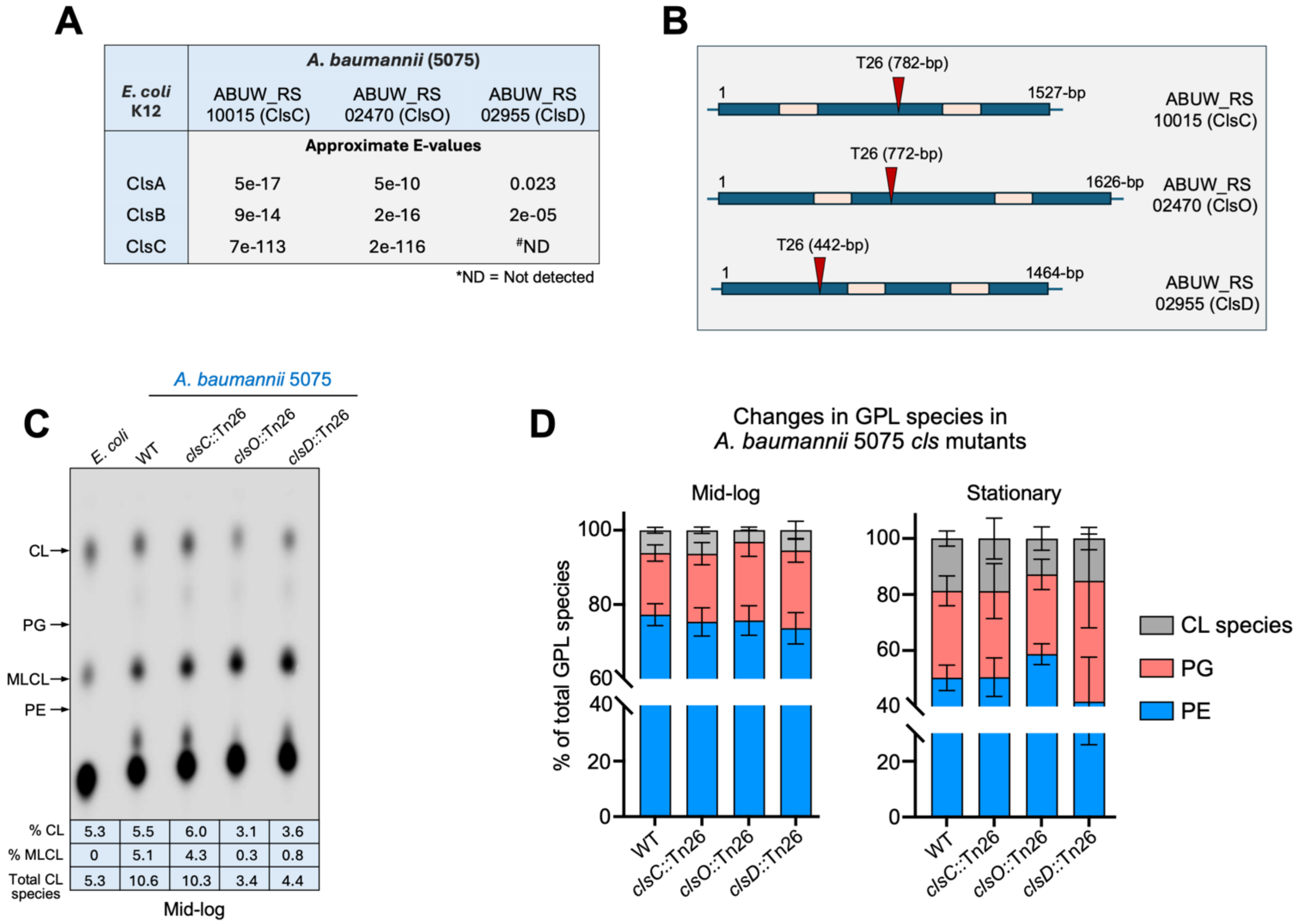

Fig. S4. Analysis of CL synthases of *A. baumannii* strain 5075. (A) Homology of putative CL synthases in *A. baumannii* 5075 to *E. coli* ClsA, B, and C. **(B)** Location of Tn-insertions in *cls* genes (*ABUW_RS10015, 02470*, and *02995*) in individual 5075 mutants from an ordered Tn-library. **(C)** ^{32}P-labeled GPLs from the indicated 5075 mutants in mid-log were isolated and analyzed by TLC. The percentage of CL species produced in each strain is given and TLC image is representative of a minimum of three biological replicates. **(D)** Quantification of GPL species in 5075 single *cls* mutants in both mid-log and stationary phase cultures from biological triplicates. Inactivation of *clsO* resulted in the largest decrease in CL species.

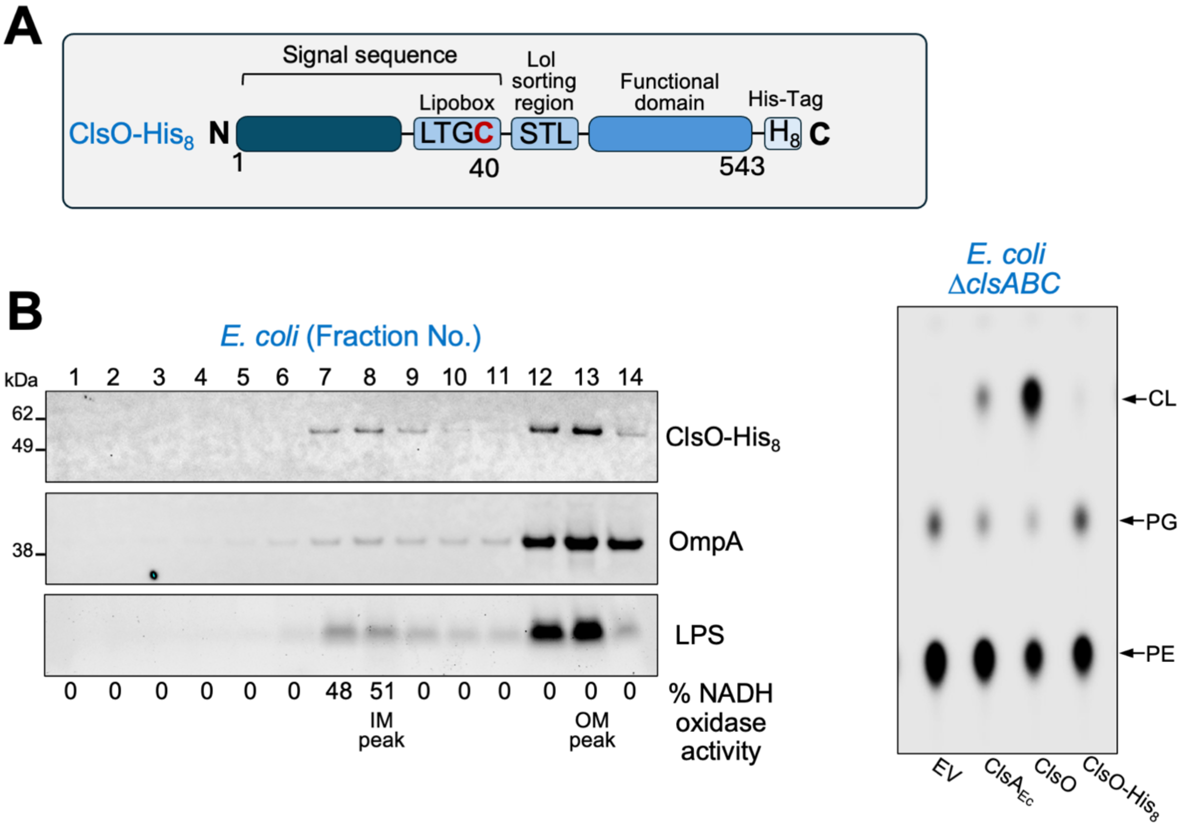

Fig. S5. C-terminally His-tagged ClsO localizes to the OM but does not retain enzymatic activity. (A) Schematic highlighting the lipoprotein signal peptide of ClsO containing the lipobox motif and the putative Lol sorting region. The N-terminal, lipidated cysteine residue of the mature protein is shown in red. To visualize the protein for membrane separation studies, a His$_8$-tag was inserted at the C-terminal end of the protein. **(B)** OM and IM fractions of *E. coli* expressing ClsO-His$_8$ from pBAD18 (0.02% arabinose) were separated by isopycnic sucrose density gradient centrifugation. Presence of ClsO-His$_8$ was evaluated by western blot. OM fractions were determined by detection of both LPS and the presence of OmpA and IM fractions determined by NADH oxidase activity (% of total). Following growth in the presence of $^{32}P_i$, GPLs from the indicated strains were isolated and analyzed by TLC. *E. coli ΔclsABC* expressing ClsA$_{Ec}$ was included as a positive control. CL in cells expressing C-terminally tagged ClsO was barely visible. Data in Panels B and C are representative of three and two biological replicates, respectively.

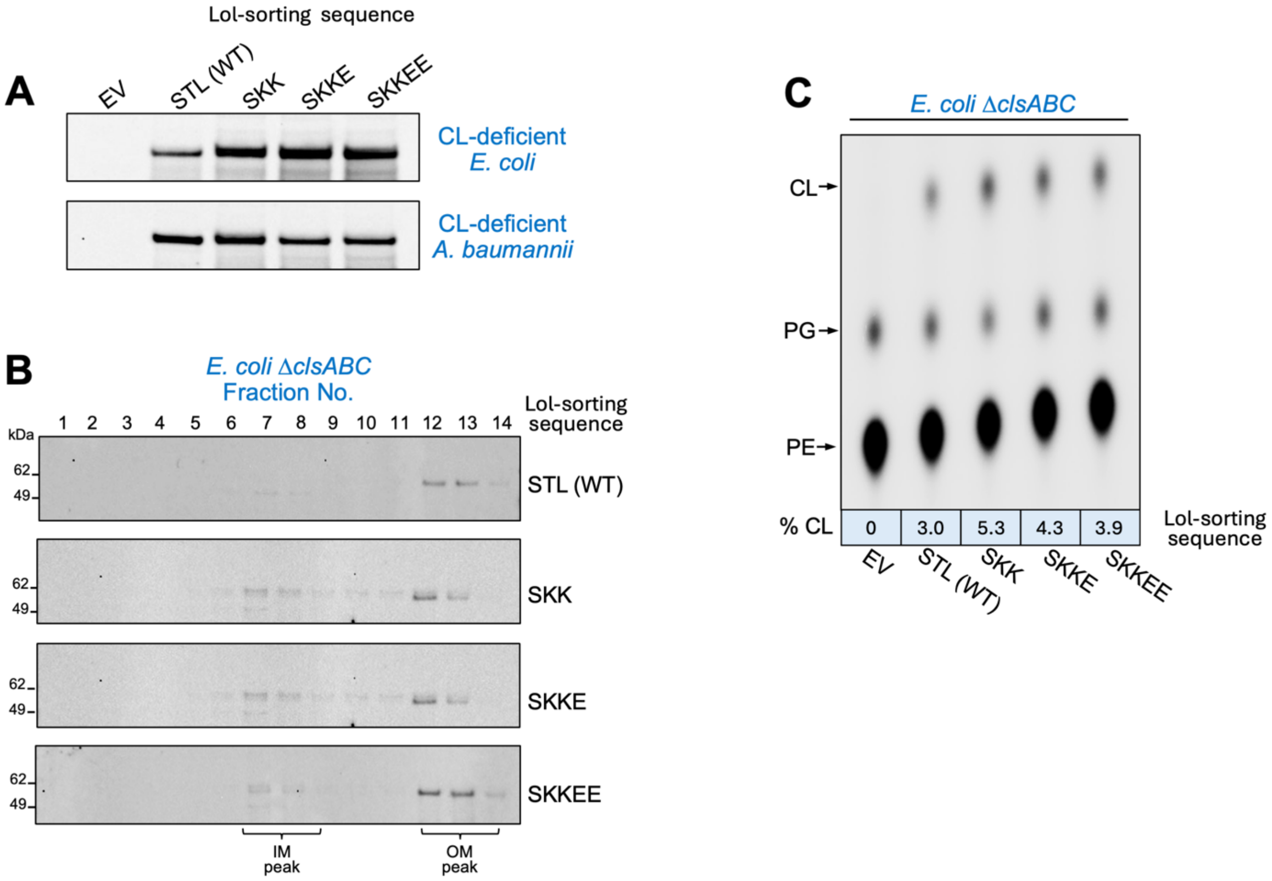

Fig. S6. Membrane localization of ClsO variants with altered Lol-sorting signal peptides in *E. coli*. **(A)** Western blot showing expression of Lol-sorting variants, described in **Fig 7**, from plasmid pMMB67EH in both CL-deficient *E. coli* (10 μM IPTG induction) and CL-deficient *A. baumannii* (50 μM IPTG induction). Each lane was loaded with 2 μg of crude cell lysate. **(B)** His_8-ClsO Lol-sorting variants described were expressed in CL-deficient *E. coli* and the IM and OM fractions separated by sucrose density gradient. ClsO was detected by Western blot. IM and OM peak fractions were determined as described in **Fig 7**. **(C)** Analysis of ^{32}P-labeled GPLs isolated from *E. coli* Δ*clsABC* expressing ClsO variants. The level of CL production was quantified by phosphorimager analysis and is indicated. Unlike what was observed in *A. baumannii*, all variants localized primarily to the OM when expressed in *E. coli* and retained enzymatic activity. Data in panels A and B are representative of two biological replicates and data in panel C representative of three biological replicates.

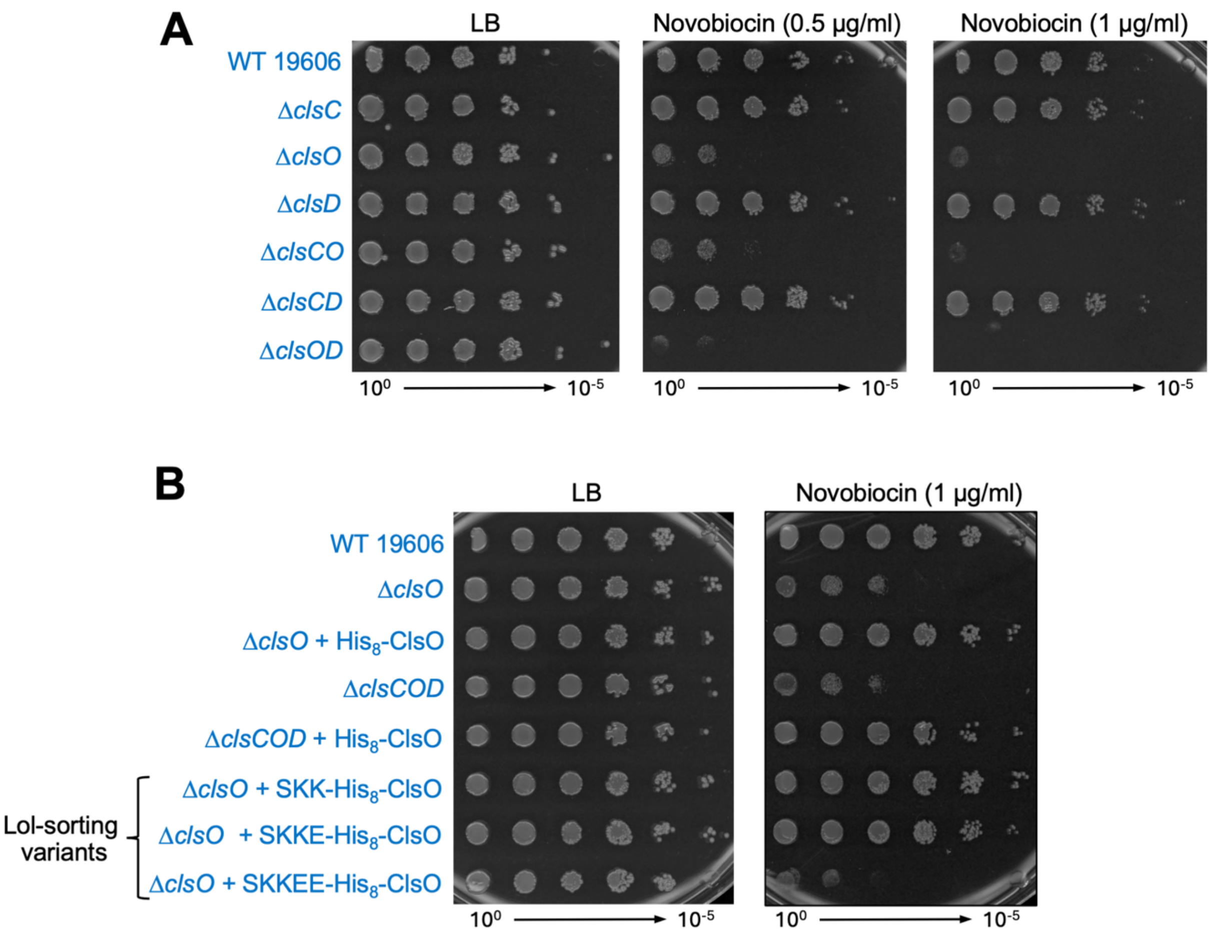

Fig. S7. ClsO is important for resistance to novobiocin. (A) Serial dilutions of the indicated CL synthases mutants were spotted on LB plates or LB plates containing either 0.5 µg/ml or 1 µg/ml novobiocin and grown at 37ºC. **(B)** Novobiocin resistance of CL-deficient *A. baumannii* expressing ClsO Lol-sorting variants. Data are representative of a minimum of three biological replicates.

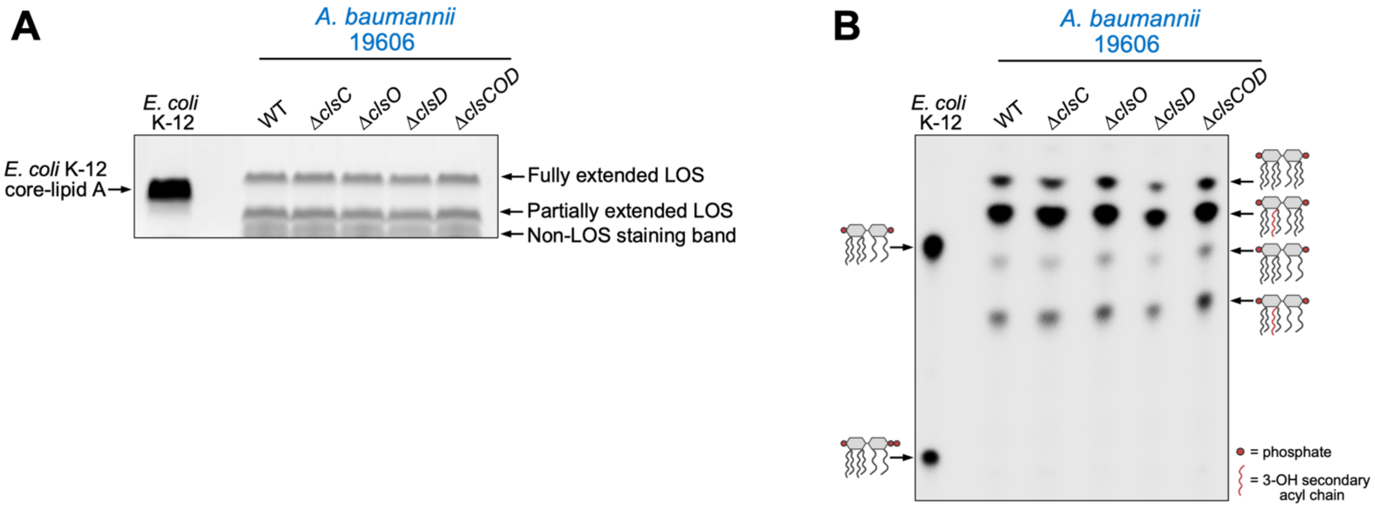

Fig. S8. Loss of ClsO has no impact on LOS structure or synthesis. (A) Changes in *A. baumannii* LOS structure and/or synthesis was determined by staining LOS after SDS-PAGE of proteinase K-treated whole cell lysates of *cls* mutants. LPS from *E. coli* K-12 strain W3110 was included as a control. As expected, *A. baumannii* produced LOS species with both partially and fully extended core oligosaccharides. **(B)** Qualitative changes in lipid A structure in mutants lacking CL synthases were assessed. Cells were grown in the presence of $^{32}P_i$, the lipid A domain of LOS isolated, and the samples analyzed by TLC. *E. coli* K12 strain W3110 was included as a control and produced the expected *bis*- and *tris*-phosphorylated, hexa-acylated lipid A species. Compared to wild type, loss of CL synthases had no impact on *A. baumannii* lipid A structure. The typical hexa- and hepta-acylated species were detected. Data in Panels A and B are representative of three and two biological replicates, respectively.

A. baumannii protein	*E. coli* Cls enzyme	BLASTp Score	E-value	Identity (%)	Positives (%)	Gaps (%)	Coverage (%)	Structural Alignment Score	*RMSD
HMPREF 0010_00607	ClsA	70.5	3e-17	20	39	21	78	417	206 pairs: 1.03 Å
	ClsB	58.9	9e-14	22	37	20	73	478.5	219 pairs: 1.01 Å
	ClsC	327	1e-111	39	57	2	91	1206.1	360 pairs: 0.93 Å
HMPREF 0010_03706	ClsA	122	5e-10	23	43	7	57	445.3	223 pairs: 0.90 Å
	ClsB	65.5	8e-16	22	39	20	75	468.8	218 pairs: 0.88 Å
	ClsC	339	1e-115	38	57	2	88	1262.4	362 pairs: 0.91 Å
HMPREF 0010_02731	ClsA	22.7	0.024	25	57	14	11	355.6	160 pairs: 1.00 Å
	ClsB	64.7	2e-05	25	42	19	45	359.5	178 pairs: 1.05 Å
	ClsC	#ND	ND	ND	ND	ND	ND	258.5	137 pairs: 0.98 Å

Table S1 Comparison of putative *A. baumannii* CL synthases with *E. coli* CL enzymes. Sequence comparisons made using BLASTp (22). *RMSD: aligned amino acid pairs with a distance of 2.0 Å or greater were removed from RMSD calculation. RMSD calculations generated using ChimeraX (20). #ND indicates not detected.

Organism	Protein ID	BlastP score	% Identity	Lipo-signal sequence score (SignalP-5.0 / 6.0)	Lipoprotein signal sequence
Acinetobacter baumannii 19606	EEX01694.1	NA	100	0.80/0.74	MIMAQSFHSKQLQTHQLAKGFLIKASIVVCSSFAVALTGCSTLPKH
Acinetobacter baumannii 5075	WP_000079186.1	1111	99.1	0.83/0.95	MAQSFHSKQLQTHQLAKGFLIKASIVVCSSFAVALTGCSTLPKH
Acinetobacter baumannii 17978	WP_000079184.1	1115	99.5	0.83/0.95	MAQSFHSKQLQTHQLAKGFLIKASIVVCSSFAVALTGCSTLPKH
Acinetobacter baylyi	WP_081398986.1	860	79.8	1.0/1.0	MTKQHAIRHCMLTLSLVTVVGINGCSTLPKH
Acinetobacter nosocomialis	WP_022575185.1	1087	96.1	0.88/0.95	MAQSFHSKQLQTHQQAKGFLIKASLIVCSSFAVTLTGCSTLPKH
Burkholderia diffusa	WP_174908651.1	333	37.7	1.0/1.0	MITLRSWSAILSLLMLAACASLPPQ
Paraburkholderia graminis	WP_044020461.1	345	37.7	0.99/1.0	MMNLRSLVAAIVVVLLTSCASLPPQ
Pseudomonas segetis	WP_141133424.1	345	38.1	0.96/0.96	MLRRFLLLALLALSGCASTPPP
Shewanella algae	WP_144165921.1	321	34.9	0.98/1.0	MLPQFGILLILLSLLSACSSAPTR
Vibrio parahaemolyticus	WP_005479787.1	296	34.6	1.0/1.0	MLHTLSKFIFASMFSVLSACSSIESN
Psychrobacter cryohalolentis	WP_011513144.1	309	35.2	1.0/1.0	MSMFSIEPSNLSLSLTLGLTLGLSGCQSLPKQ
Helicobacter pylori	WP_000689051.1	221	31.6	0.98/0.65	MKIFLVFLSVFFFNGCFGLVYK
Cupriavidus pauculus	WP_244789155.1	339	37.7	1.0/1.0	MVMVTLTLGLPAGCASLPAN
Moraxella catarrhalis	WP_003657922.1	326	35.4	1.0/1.0	MTHHFRSLFVISIHTVMLVLLVGCKNLPDT
Neisseria gonorrhoeae	WP_010358244.1	216	49.2	1.0/1.0	MKTRSLISLLCLLLCSCSSWLPP
Bordetella pertussis	WP_023853368.1	346	38.4	1.0/1.0	MPRLLSRIRAARARAAALLALAGCAILLGACTLPPPV
Snodgrassella alvi	WP_370389071.1	363	37.3	1.0/1.0	MNSKIFNQICTICFKLCTLFILTACQTLPSL

Table S2: Lipoprotein prediction of representative bacterial species with a ClsO homolog. For each ClsO homolog the predicted lipoprotein signal sequence is shown. The lipoprotein signal sequence was predicted using both SignalP 5.0 and 6.0 (16, 17). The lipobox is underlined and the putative sorting signal is shown in italics. The modified, acylated cysteine residue is shown in red. Note that the score for the *Helicobacter pylori* ClsO is 0.65 using SignalP 6.0, but is 0.98 using SignalP version 5.0.

Gene of interest	Early stationary vs. logarithmic		Late stationary vs. logarithmic	
***E. coli* K12 (W3110)**	**Fold Change**	**P-value**	**Fold Change**	**P-value**
clsA	-1.01	0.93	1.38	0.05
clsB	4.96	0	9.33	0
clsC	1.43	0.01	2.3	6.45E-09
***A. baumannii* (19606)**	**Fold Change**	**P-value**	**Fold Change**	**P-value**
HMPREF0010_00607 (*clsC*)	-1.12	0.63	-1.4	0.06
HMPREF0010_03706 (*clsO*)	-1.25	0.37	-1.54	0.03
HMPREF0010_02731(*clsD*)	-1.15	0.57	1.06	0.76

Table S3. Expression of CL synthases in *E. coli* K12 and *A. baumannii* at different phases of growth. Weighted fold changes from triplicate RNA-seq at different growth stages in LB media are shown. Data shown is for wild-type *E. coli* strain W3110 and wild-type *A. baumannii* strain 19606.

Annotated peptide	Peptide modification	No. of peptide-spectrum matches	Confidence	Peptide in mature ClsO	Residue in mature ClsO
[R].DIDTSQTSLSKIITPLR.[E]	1xNHS-PEG$_{12}$-Biotin [K11]	1	High	17-33	K27
[R].EKNPNLTGYHLLNDPLEALAAR.[L]	1xNHS-PEG$_{12}$-Biotin [K2]	3	High	44-65	K35
[R].DLNKNTDELATK.[A]	1xNHS-PEG$_{12}$-Biotin [K4]	2	High	376-387	K379
[K].NTDELATKAK.[V]	1xNHS-PEG$_{12}$-Biotin [K8]	1	High	380-389	K387
[R].SAYLNTEIGVILDSPSLAKTIHHTMDENLNK.[Y]	1xNHS-PEG$_{12}$-Biotin [K19]	1	High	422-452	K440
[K].TIHHTMDENLNKYAYK.[L]	1xNHS-PEG$_{12}$-Biotin [K12]	2	High	441-456	K452

Table S4. Identification of ClsO residues modified by NHS-[PEG]$_{12}$-biotin. Intact *A. baumannii* expressing His-tagged ClsO were exposed to NHS-[PEG]$_{12}$-biotin and modified lysine residues identified by proteomics mass spectrometry. Peptides containing solvent-exposed, modified lysine residues are shown along with the position of each lysine within the protein.

Supplemental References

1. P. D. Karp, *et al.*, The BioCyc collection of microbial genomes and metabolic pathways. *Brief Bioinform* **20**, 1085–1093 (2019).
2. L. A. Gallagher, *et al.*, Resources for Genetic and Genomic Analysis of Emerging Pathogen Acinetobacter baumannii. *J. Bacteriol.* **197**, 2027–2035 (2015).
3. A. T. Tucker, *et al.*, Defining gene-phenotype relationships in Acinetobacter baumannii through one-step chromosomal gene inactivation. *MBio* (2014).
4. K. A. Datsenko, B. L. Wanner, One-step inactivation of chromosomal genes in Escherichia coli K-12 using PCR products. *Proc. Natl. Acad. Sci. U.S.A.* **97**, 6640–6645 (2000).
5. R. de Dios, K. Gadar, R. R. McCarthy, A high-efficiency scar-free genome-editing toolkit for Acinetobacter baumannii. *J Antimicrob Chemother* **77**, 3390–3398 (2022).
6. R. M. Horton, H. D. Hunt, S. N. Ho, J. K. Pullen, L. R. Pease, Engineering hybrid genes without the use of restriction enzymes: gene splicing by overlap extension. *Gene* **77**, 61–68 (1989).
7. M. V. Douglass, F. Cléon, M. S. Trent, Cardiolipin aids in lipopolysaccharide transport to the gram-negative outer membrane. *Proc Natl Acad Sci U S A* **118** (2021).
8. M. J. Powers, B. W. Simpson, M. S. Trent, The Mla pathway in Acinetobacter baumannii has no demonstrable role in anterograde lipid transport. *Elife* **9** (2020).
9. C. M. Herrera, B. J. Voss, M. S. Trent, Homeoviscous Adaptation of the Acinetobacter baumannii Outer Membrane: Alteration of Lipooligosaccharide Structure during Cold Stress. *mBio* **12**, e0129521 (2021).
10. M. J. Powers, C. M. Herrera, A. T. Tucker, B. W. Davies, M. S. Trent, Isolation of Lipid Cell Envelope Components from Acinetobacter baumannii. *Methods Mol. Biol.* **1946**, 233–252 (2019).
11. A. C. Pride, C. M. Herrera, Z. Guan, D. K. Giles, M. S. Trent, The outer surface lipoprotein VolA mediates utilization of exogenous lipids by Vibrio cholerae. *MBio* **4**, e00305-00313 (2013).
12. R. Shrivastava, X. Jiang, S.-S. Chng, Outer membrane lipid homeostasis via retrograde phospholipid transport in Escherichia coli. *Mol Microbiol* **106**, 395–408 (2017).
13. M. B. Cian, N. P. Giordano, J. A. Mettlach, K. E. Minor, Z. D. Dalebroux, Separation of the Cell Envelope for Gram-negative Bacteria into Inner and Outer Membrane Fractions with Technical Adjustments for Acinetobacter baumannii. *J Vis Exp* (2020). https://doi.org/10.3791/60517.
14. M. S. Trent, W. Pabich, C. R. Raetz, S. I. Miller, A PhoP/PhoQ-induced Lipase (PagL) that catalyzes 3-O-deacylation of lipid A precursors in membranes of Salmonella typhimurium. *The Journal of biological chemistry* **276**, 9083–92 (2001).
15. L. M. VanOtterloo, L. A. Macias, M. J. Powers, J. S. Brodbelt, M. S. Trent, Characterization of Acinetobacter baumannii core oligosaccharide synthesis reveals novel aspects of lipooligosaccharide assembly. *mBio* e0301323 (2024). https://doi.org/10.1128/mbio.03013-23.
16. F. Teufel, *et al.*, SignalP 6.0 predicts all five types of signal peptides using protein language models. *Nat Biotechnol* **40**, 1023–1025 (2022).
17. J. J. Almagro Armenteros, *et al.*, SignalP 5.0 improves signal peptide predictions using deep neural networks. *Nat Biotechnol* **37**, 420–423 (2019).
18. C. J. A. Sigrist, *et al.*, New and continuing developments at PROSITE. *Nucleic Acids Res* **41**, D344-347 (2013).
19. J. Abramson, *et al.*, Accurate structure prediction of biomolecular interactions with AlphaFold 3. *Nature* **630**, 493–500 (2024).

20. E. C. Meng, *et al.*, UCSF ChimeraX: Tools for structure building and analysis. *Protein Sci* **32**, e4792 (2023).
21. I. Letunic, P. Bork, Interactive Tree Of Life (iTOL) v5: an online tool for phylogenetic tree display and annotation. *Nucleic Acids Res* **49**, W293–W296 (2021).
22. E. W. Sayers, *et al.*, Database resources of the National Center for Biotechnology Information in 2025. *Nucleic Acids Res* **53**, D20–D29 (2025).
